# Supplementary material for: 2.5-dimensional covalent organic frameworks
Source: Nat Commun. 2025 Jan 2;16:280. doi: 10.1038/s41467-024-55729-2 (PMC11696810; doi:10.1038/s41467-024-55729-2)
Supplement: Supplementary file 1 — Supplementary Information [file 41467_2024_55729_MOESM1_ESM.pdf]

Supplementary Information

2.5-dimensional covalent organic frameworks

Tomoki Kitano[1,2], Syunto Goto[1,2], Xiaohan Wang[1,2], Takayuki Kamihara[3], Yoshihisa Sei[3], Yukihito Kondo[4], Takumi Sannomiya[4], Hidehiro Uekusa[5] and Yoichi Murakami[1,2,6*]

[1] Laboratory for Zero-Carbon Energy, Institute of Integrated Research, Institute of Science Tokyo[†], Tokyo, Japan.

[2] Department of Mechanical Engineering, Institute of Science Tokyo[†], Tokyo, Japan.

[3] Facility Station Division, Open Facility Center, Institute of Science Tokyo[†], Yokohama, Japan.

[4] Department of Materials Science & Engineering, Institute of Science Tokyo[†], Yokohama, Japan.

[5] Department of Chemistry, Institute of Science Tokyo[†], Tokyo, Japan.

[6] Department of Transdisciplinary Science & Engineering, Institute of Science Tokyo[†], Tokyo, Japan.

*e-mail: murakami.y.af@m.titech.ac.jp

[†]Tokyo Institute of Technology before October 1, 2024.

Table of Contents

Section S1. List of synthetic conditions of COFs used for each data (Table S1)

Section S2. Results of supplementary characterizations

S2.1 Elemental analyses (EA) (Table S2)

S2.2 FT-IR spectra (Fig. S1)

S2.3 Solid-state ^{13}C CP/MAS NMR spectra (Fig. S2)

S2.4 XPS spectra (Fig. S3)

S2.5 PXRD patterns (Figs. S4 and S5)

S2.6 AFM images of **TK-COF-5** (Fig. S6)

S2.7 Structure of **TK-COF-4** determined by SCXRD (Figs. S7 and S8, Table S3)

S2.8 Structure of **TK-COF-5** determined by SCXRD (Figs. S9−S12, Table S4)

S2.9 Determination of crystal face indexes by SCXRD measurements (Figs. S13 and S14)

S2.10 Framework densities of **TK-COF-4/-5** and hypothetical 3D-COFs with **bor** and **ctn** topology (Table S5)

S2.11 Area densities of primary amines of **TK-COF-4/-5** and other COFs (Table S6)

S2.12 Effect of solvent removal on crystal structure (Figs. S15−S17, Table S7)

S2.13 Assessment of thermal stability (Figs. S18−S21)

S2.14 Results of gas adsorption measurements and analyses (Figs. S22−S26, Table S8)

S2.15 Advantage in CO_2 adsorption rate of the highly crystalline material over the reference material with lower crystallinity (Figs. S27−S34)

S2.16 Energy calculations of the frameworks (Tables S9 and S10)

Section S3. CO_2 adsorption properties reported for previous COFs, MOFs, and POPs

S3.1 COFs (Table S11)

S3.2 MOFs (Fig. S35)

S3.3 POPs (Fig. S36)

Section S4. Explanation for A- and B-level alerts in checkCIF report

References

Section S1. List of synthetic conditions of COFs used for each data

Supplementary Table S1. Summary of samples used in this article.

Figure #	Purpose	Type of COF	Preparation condition	Time for growth	Sample state
Fig. 1e	Optical microscopy	**TK-COF-4**	I	6 days	In *o*-dichlorobenzene (*o*-DCB)
		**TK-COF-5**	III	11 days	
Fig. 1f	Solid-state ^{15}N CP/MAS NMR	**TK-COF-4**	II	3 days	Dried, in dry air
		**TK-COF-5**	IV	4 days	
Fig. 1g	PXRD	**TK-COF-4**	II	2 days	In acetonitrile
		**TK-COF-5**	IV	3 days	
Fig. 1h	SEM	**TK-COF-4**	II	2 days	Dried, in vacuum
		**TK-COF-5**	III	6 days	
Fig. 1i	AFM	**TK-COF-4**	II	2 days	Dried, in air
Fig. 2a	SCXRD	**TK-COF-4**	I	7 days	In a mixture of *o*-DCB, $[N_{8881}][NTf_2]$, and *CryoOil*
		**TK-COF-5**	III	6 days	
Fig. 2b	HR-TEM	**TK-COF-4**	II	7 days	Dried, in vacuum
Fig. 2c		**TK-COF-5**	IV	6 days	
Fig. 3a	TGA	**TK-COF-4**	II	3 days	In air or N_2 flow
		**TK-COF-5**	IV	4 days	
Fig. 3b	PXRD	**TK-COF-4**	II	3 days	Dried, in air
		**TK-COF-5**	IV	4 days	
Fig. 3c	Gas adsorption	**TK-COF-4**	II	3 days	Dried
Fig. 3d		**TK-COF-5**	IV	4 days	
Table S2	EA	**TK-COF-4**	II	3 days	Dried
		**TK-COF-5**	IV	4 days	
Fig. S1	FT-IR	**TK-COF-4**	II	2 days	Dried, in vacuum
		**TK-COF-5**	IV	4 days	

Fig. S2	Solid-state ^{13}C CP/MAS NMR	**TK-COF-4**	II	3 days	Dried, in dry air
		**TK-COF-5**	IV	4 days	
Fig. S3	XPS	**TK-COF-4**	II	3 days	Dried, in vacuum
		**TK-COF-5**	IV	4 days	
Fig. S4	PXRD	**TK-COF-4**	II	3 days	Dried, in air
		**TK-COF-5**	IV	4 days	
Fig. S5		**TK-COF-4**	I	8 days	In acetonitrile
			II	2 days	
		**TK-COF-5**	III	2 days	
			IV	5 days	
Fig. S6	AFM	**TK-COF-5**	IV	3 days	Dried, in air
Fig. S7	SCXRD	**TK-COF-4**	I	7 days	In a mixture of *o*-DCB, $[N_{8881}][NTf_2]$, and *CryoOil*
Fig. S8	PXRD	**TK-COF-4**	I	8 days	In acetonitrile
Fig. S9	SCXRD	**TK-COF-5**	III	6 days	In a mixture of *o*-DCB, $[N_{8881}][NTf_2]$, and *CryoOil*
Fig. S10	PXRD	**TK-COF-5**	III	5 days	In acetonitrile
Fig. S11			IV	4 days	In *o*-DCB or acetonitrile
Fig. S12			IV	4 days	In *o*-DCB or toluene
Fig. S13	Determination of crystal face indexes	**TK-COF-4**	I	11 days	In a mixture of *o*-DCB and $[N_{8881}][NTf_2]$
Fig. S14		**TK-COF-5**	III	11 days	
Fig. S15	SCXRD	**TK-COF-5_dried**	III	6 days	Dried, in oil
Fig. S16	PXRD	**TK-COF-5**	IV	4 days	Dried, in air
Fig. S18		**TK-COF-4**	II	3 days	Dried, in air
Fig. S19		**TK-COF-5**	IV	4 days	
Fig. S20	CO_2 adsorption	**TK-COF-4**	II	4 days	Dried
Fig. S21a	PXRD	**TK-COF-4**	II	4 days	Dried
Fig. S21b	FT-IR	**TK-COF-4**	II	4 days	Dried, in vacuum
Figs. S24, S26	CO_2 adsorption	**TK-COF-4**	II	3 days	Dried
		**TK-COF-5**	IV	4 days	

Figs. S25, S26	N_2 adsorption	**TK-COF-4**	II	4 days	Dried
		**TK-COF-5**	IV	4 days	
Fig. S28	PXRD	**TK-COF-4**	II	2 days	In acetonitrile
Fig. S29	FT-IR	**TK-COF-4**	II	2 days	Dried, in vacuum
Figs. S30, S31, S33	Gas adsorption	**TK-COF-4**	II	4 days	Dried

Section S2. Results of supplementary characterizations

S2.1 Elemental analyses (EA)

Elemental analyses (EA) were conducted using a *Micro Corder JM10 (J-Science)*. Before EA, samples were dried in a vacuum at 80 °C for 12 h with a flow of 50 sccm of dry nitrogen. The results agreed well with the elemental compositions calculated from the results of the single-crystal X-ray diffraction (SCXRD) measurements, as summarized in Table S2 below.

Supplementary Table S2. Results of elemental analysis.

Element	**TK-COF-4** ($C_{49}H_{33}N_7$)		**TK-COF-5** ($C_{52}H_{36}N_4$)	
	Measured (wt%)	Calculated (wt%)	Measured (wt%)	Calculated (wt%)
C	79.76	81.78	85.78	87.15
H	4.91	4.59	5.30	5.03
N	13.08	13.63	7.63	7.82

S2.2 FT-IR spectra

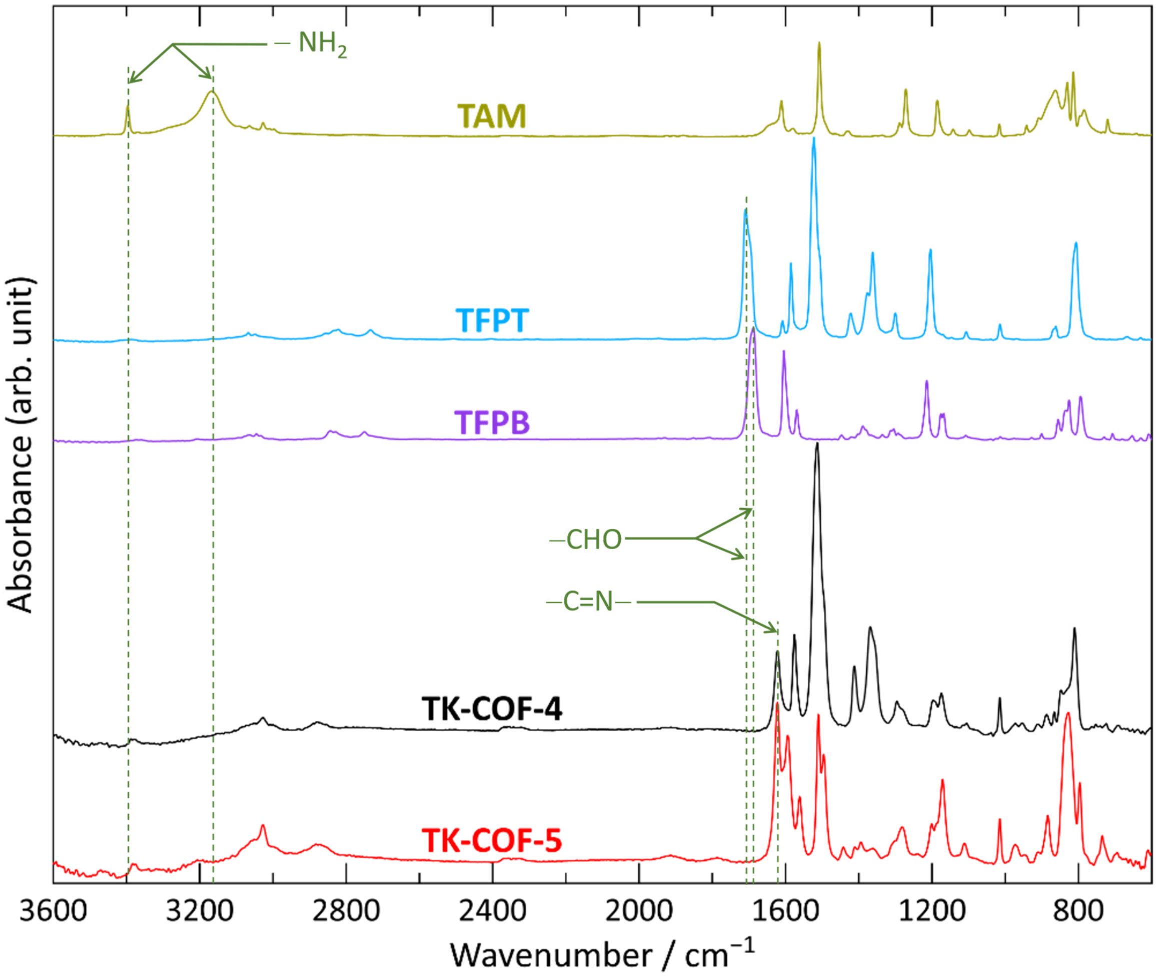

Supplementary Figure S1. FT-IR spectra of **TAM**, **TFPT**, **TFPB**, **TK-COF-4**, and **TK-COF-5**.

S2.3 Solid-state ^{13}C CP/MAS NMR spectra

Figure S2 shows solid-state ^{13}C CP/MAS NMR spectra obtained from powders of **TK-COF-4** and **-5**. We assigned the chemical shifts considering the assignments used in previous COF reports[S1–S5].

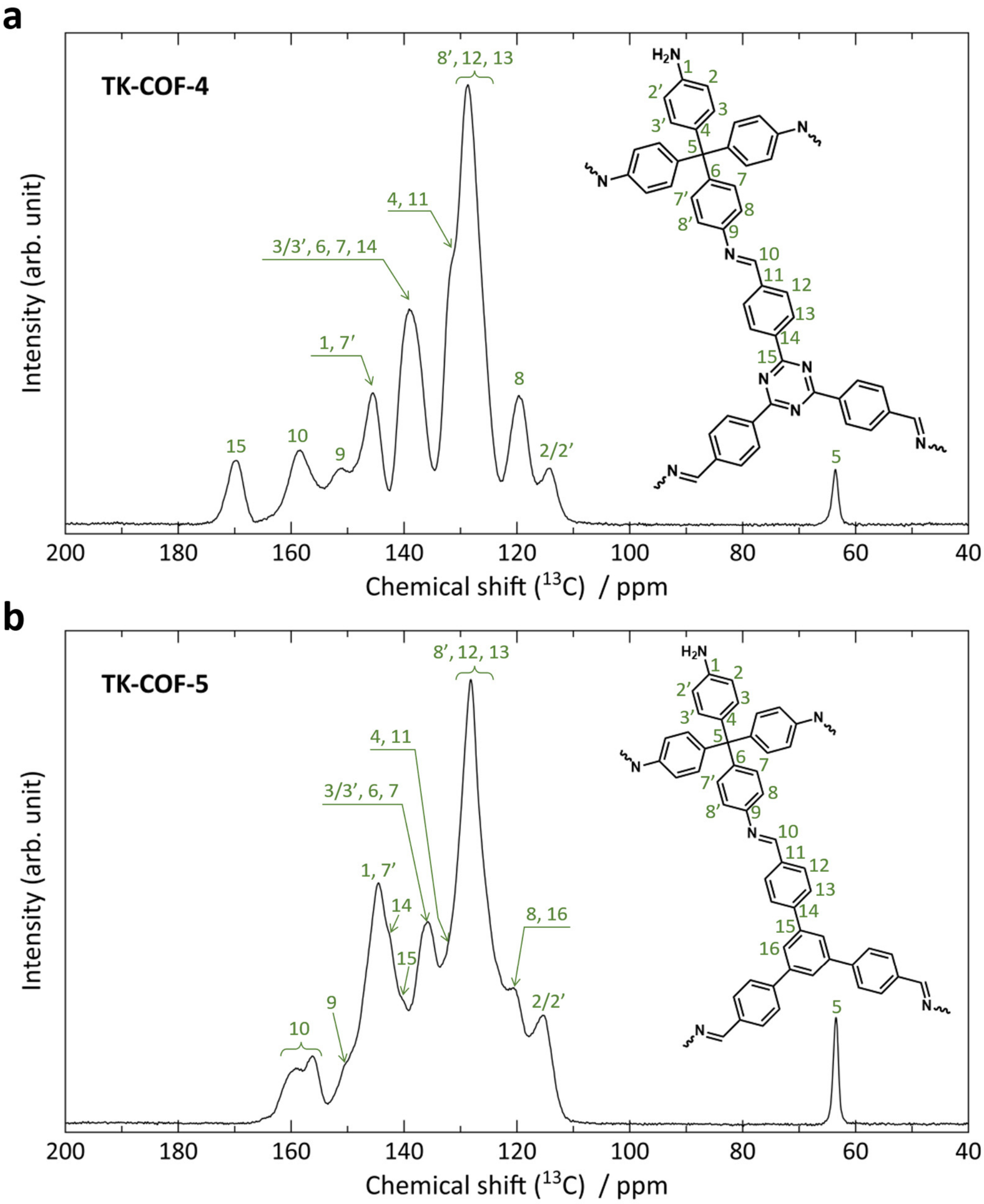

Supplementary Figure S2. Solid-state ^{13}C CP/MAS NMR spectra of **a**, **TK-COF-4**, **b**, **TK-COF-5**.

S2.4 XPS spectra

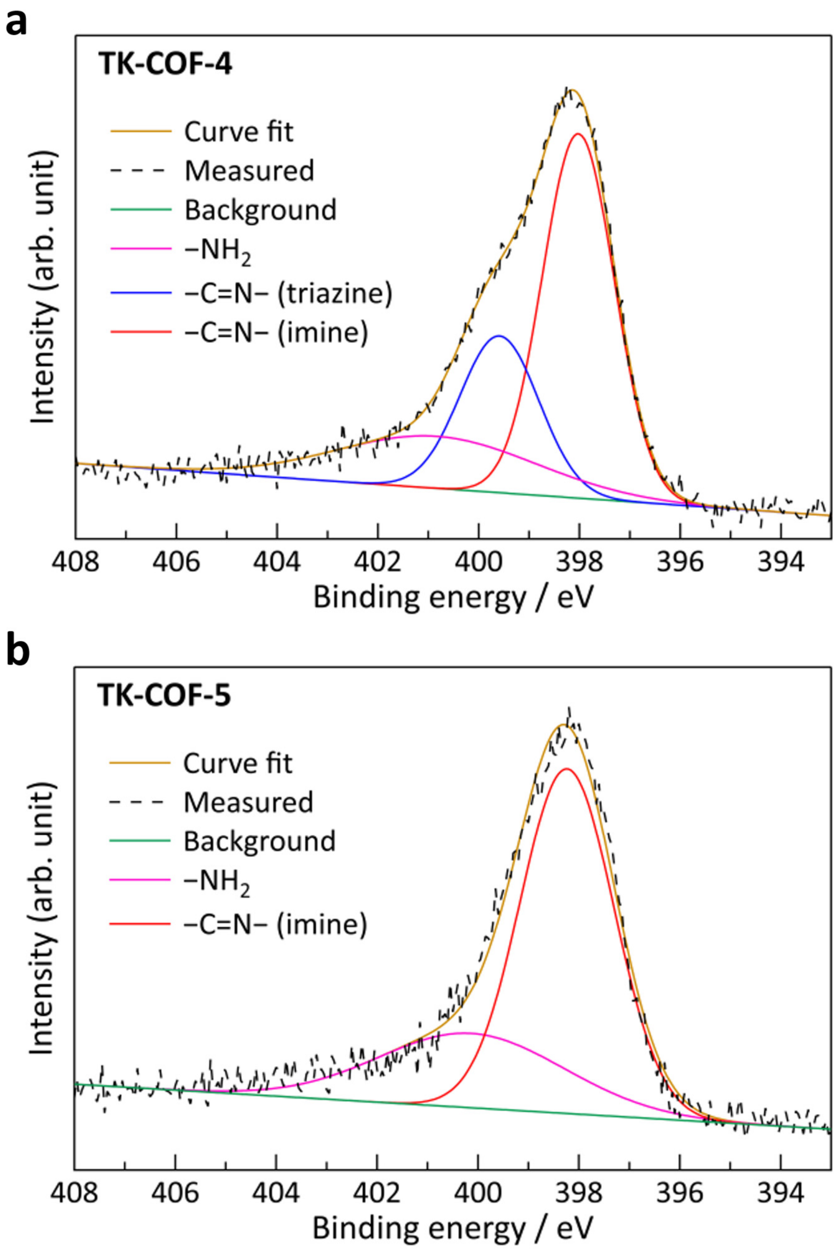

Supplementary Figure S3. XPS spectra obtained from **a**, **TK-COF-4**, **b**, **TK-COF-5**. Curve fits were conducted with Gaussian functions using *PHI MultiPak®* software.

S2.5 PXRD patterns

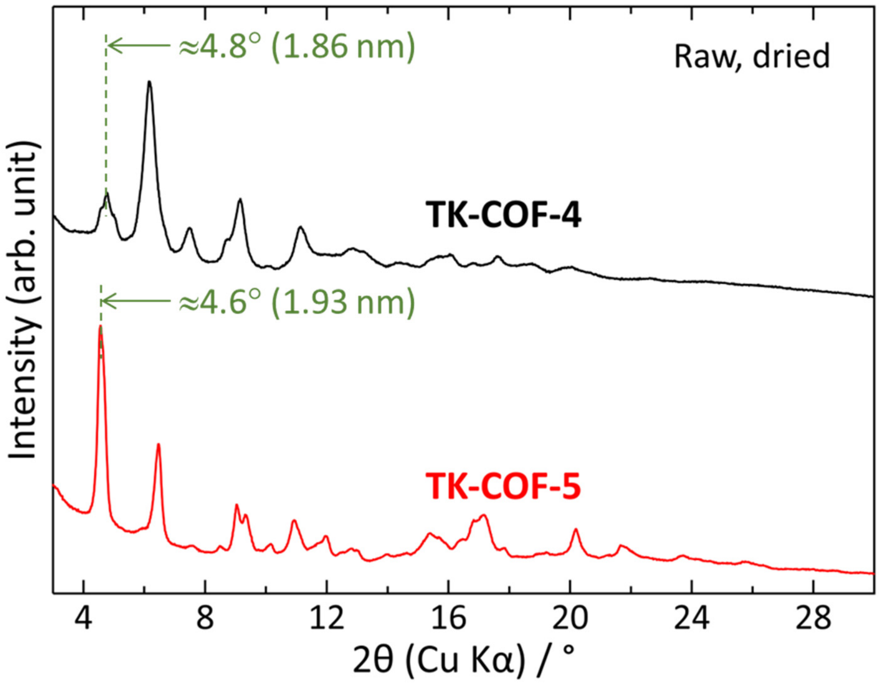

Supplementary Figure S4. PXRD patterns from **TK-COF-4** (black) and **TK-COF-5** (red) in the dried state.

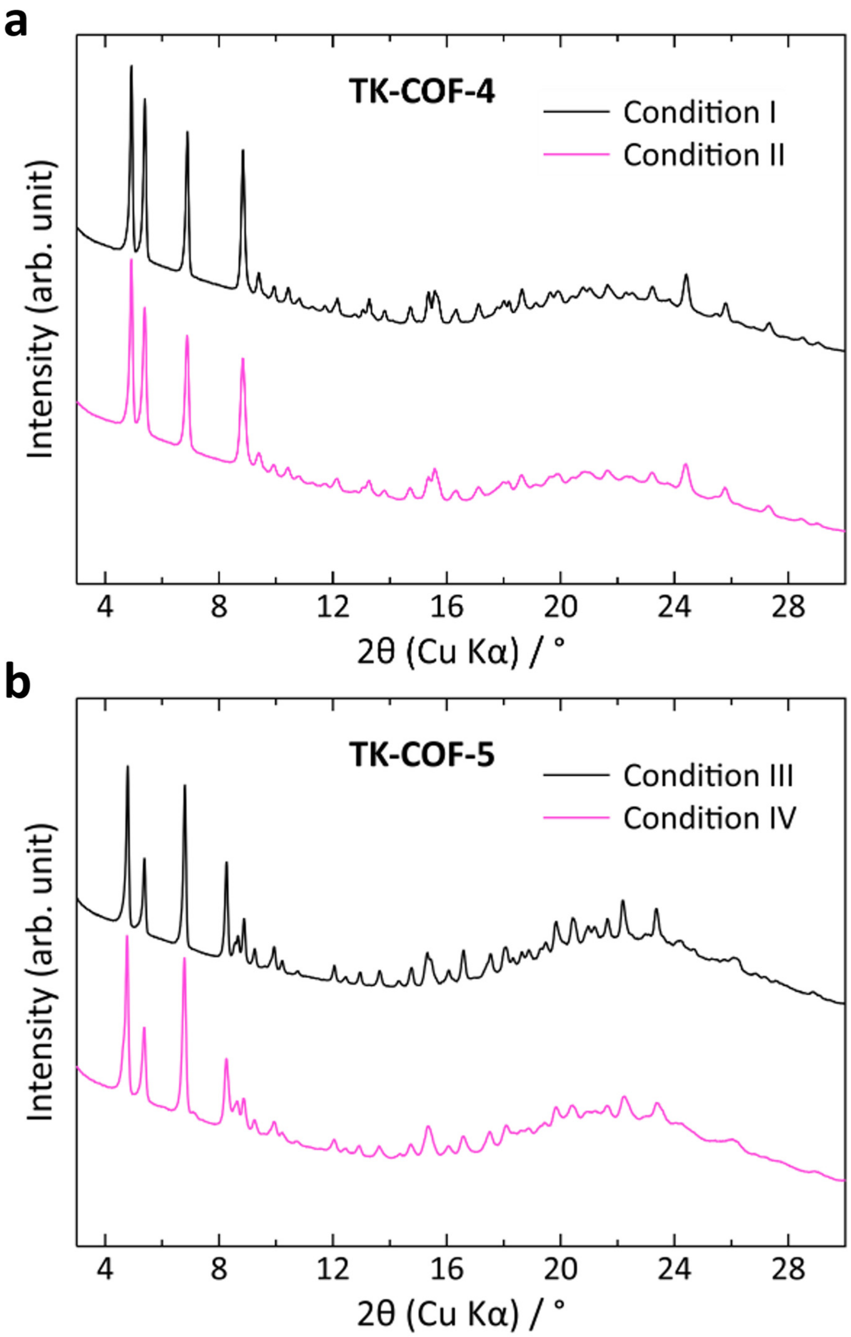

Supplementary Figure S5. Comparisons of PXRD patterns from samples grown in Conditions I and II (**a**, **TK-COF-4**) and Conditions III and IV (**b**, **TK-COF-5**) in acetonitrile.

S2.6 AFM images of TK-COF-5

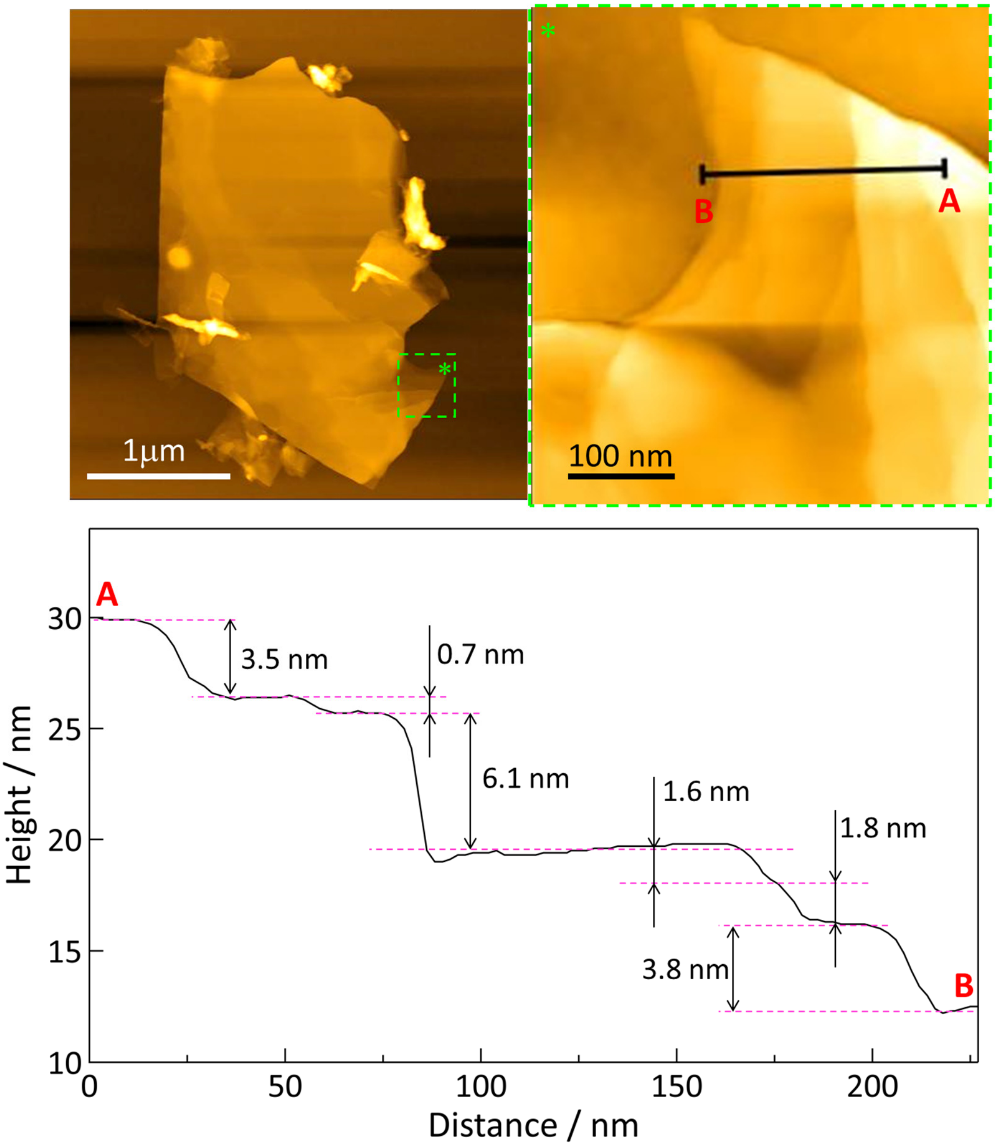

Supplementary Figure S6. AFM images of **TK-COF-5** on a silicon substrate. The sample was mechanically treated according to the method described in Methods in the main text.

S2.7 Structure of TK-COF-4 determined by SCXRD

The asymmetric unit determined is shown in Fig. S7 below. The free amine (N7) of **TAM** formed hydrogen bonding with a nitrogen of the imine moiety in an adjacent molecule, in which the distance was 2.7 Å (see also Fig. 2a in the main text). The detailed crystallographic data is shown in Supplementary Table S3.

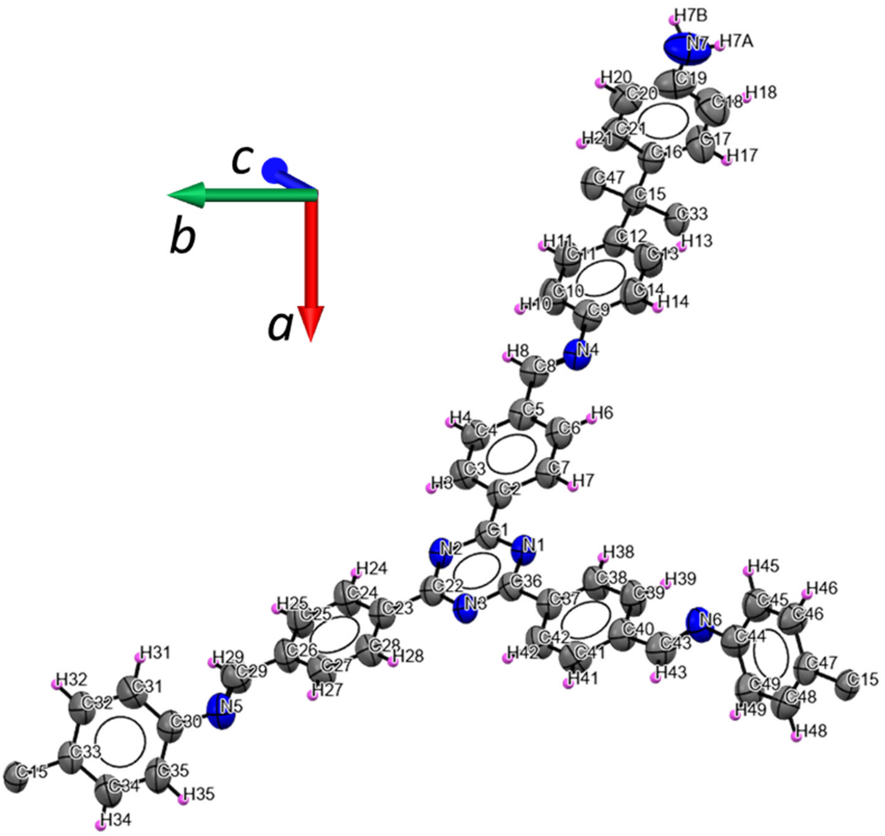

Supplementary Figure S7. Asymmetric unit of the **TK-COF-4** structure determined from SCXRD data and structural analysis. Thermal ellipsoids are drawn with 50% probability. The solvent molecules (*o*-DCB) were omitted for clarity.

Supplementary Table S3. Crystallographic data for **TK-COF-4**

**TK-COF-4**	
Chemical composition of COF	$C_{49}H_{33}N_7$
Chemical formula of guests	$Cl_4H_8C_{12}$ (two molecules of *o*-DCB)
Formula mass	1013.81
Crystal system	Monoclinic
Space group	$P2_1/n$
a, Å	9.8024(4)
b, Å	36.6977(9)
c, Å	18.2022(5)
β, °	99.608(3)
V, Å^3	6455.9(4)
d, g cm^{-3}	1.043
μ, mm^{-1}	1.963
Z	4
Reflections collected	45378
Independent reflections	13118
Observed reflections	13118
Restraints	276
Parameters	748
2θ range for data collection, °	4.816 to 153.914
Index ranges	$-12 \le h \le 11$, $-44 \le k \le 23$, $-21 \le l \le 22$
R_{int}	0.0556
R_{σ}	0.0522
Goodness-of-fit on F^2	1.250
Final R indexes $(I \ge 2\sigma(I))$	R_1 = 0.1112, wR_2 = 0.3368
Final R indexes [all data]	R_1 = 0.1568, wR_2 = 0.3718
$\Delta\rho_{max}$, e Å^{-3}	0.67
$\Delta\rho_{min}$, e Å^{-3}	−0.49
Crystal size, mm^3	0.09 × 0.08 × 0.04
Radiation, Å	Cu Kα (λ = 1.54184)
Temperature, K	93.15
CCDC number	2361003

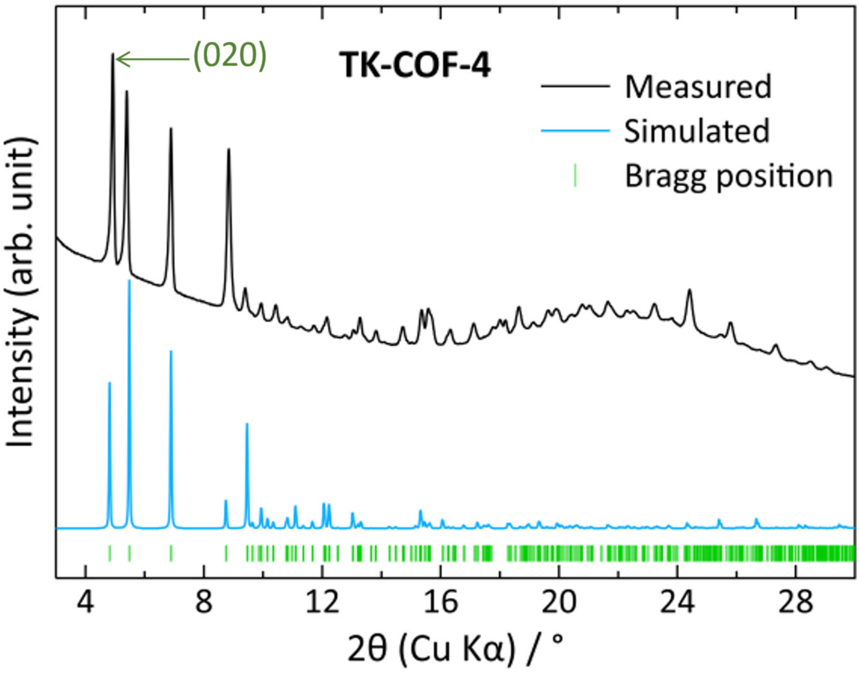

Supplementary Figure S8. Comparison of the PXRD pattern from **TK-COF-4** crystals in acetonitrile with the simulated pattern generated from the crystal structure determined by the SCXRD analysis.

S2.8 Structure of TK-COF-5 determined by SCXRD

The asymmetric unit determined is shown in Fig. S9 below. The free amine (N56) of **TAM** formed hydrogen bonding with a nitrogen of the imine moiety in an adjacent molecule, in which the distance was 2.4 Å (see also Fig. 2a in the main text). The detailed crystallographic data is shown in Supplementary Table S4.

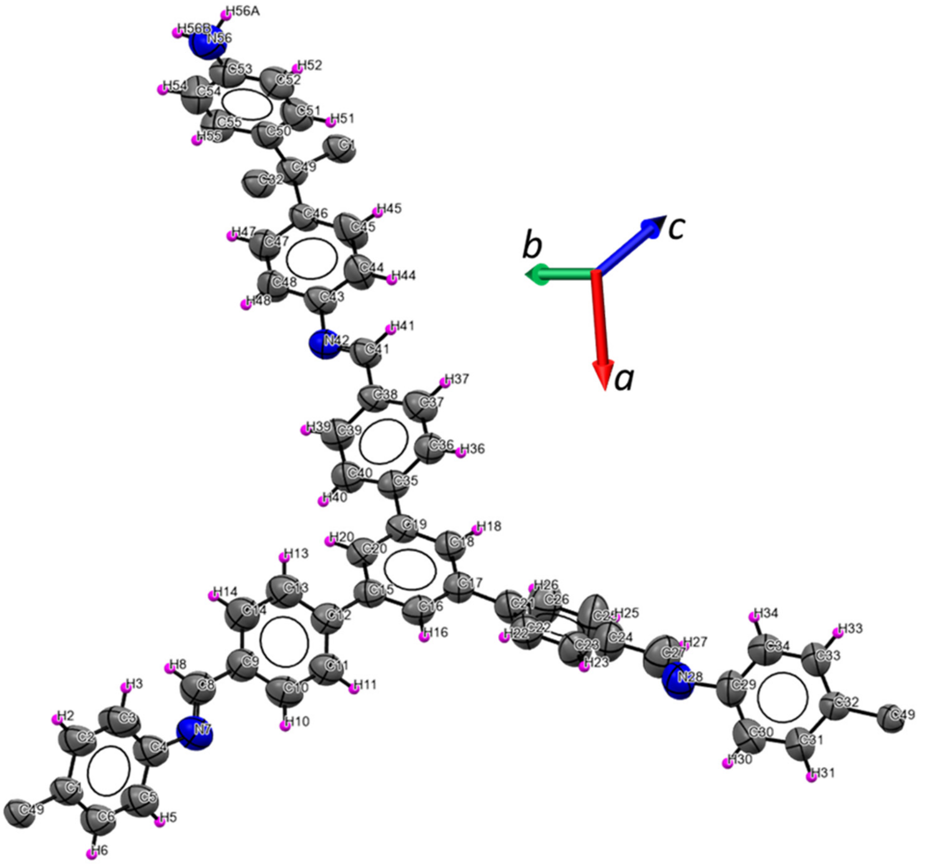

Supplementary Figure S9. Asymmetric unit of the **TK-COF-5** structure determined from SCXRD data and structural analysis. Thermal ellipsoids are drawn with 50% probability. The solvent molecules (*o*-DCB) were omitted for clarity.

Supplementary Table S4. Crystallographic data for **TK-COF-5**

**TK-COF-5**	
Chemical composition of COF	$C_{52}H_{36}N_4$
Chemical formula of guests	$Cl_6H_{12}C_{18}$ (three molecules of *o*-DCB)
Formula mass	1019.66
Crystal system	Monoclinic
Space group	$P2_1/n$
a, Å	10.2481(7)
b, Å	37.3978(18)
c, Å	18.6451(8)
β, °	103.078(5)
V, Å^3	6960.5(7)
d, g cm^{-3}	0.973
μ, mm^{-1}	1.851
Z	4
Reflections collected	43752
Independent reflections	12715
Observed reflections	12715
Restraints	271
Parameters	811
2θ range for data collection, °	4.726 to 136.498
Index ranges	$-12 \le h \le 11$, $-45 \le k \le 45$, $-10 \le l \le 22$
R_{int}	0.0781
R_σ	0.0628
Goodness-of-fit on F^2	1.347
Final R indexes ($I \ge 2\sigma(I)$)	R_1 = 0.1344, wR_2 = 0.3742
Final R indexes [all data]	R_1 = 0.1860, wR_2 = 0.4110
$\Delta\rho_{max}$, e Å^{-3}	0.93
$\Delta\rho_{min}$, e Å^{-3}	−0.42
Crystal size, mm^3	0.09 × 0.06 × 0.05
Radiation, Å	Cu Kα (λ = 1.54184)
Temperature, K	93.15
CCDC number	2361014

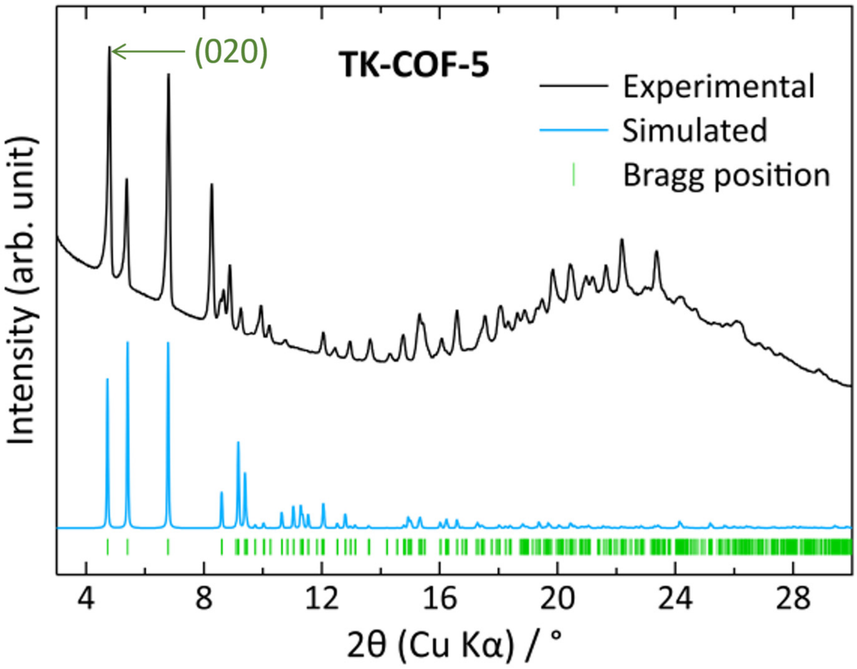

Supplementary Figure S10. Comparison of the PXRD pattern from **TK-COF-5** crystals in acetonitrile with the simulated pattern generated from the crystal structure determined by the SCXRD analysis.

In Fig. S10, we compared the PXRD pattern from **TK-COF-5** crystals in acetonitrile and the simulated pattern reconstructed from the crystal structure determined by the SCXRD measurement in *o*-DCB. Although these patterns qualitatively agree with each other, the diffraction peak at $2\theta \approx 8.3°$ is found only in the former. Below, we investigate the nature of this ≈ 8.3° peak by carrying out two series of experiments, denoted by "Series A experiments" and "Series B experiments."

In the Series A experiments, first, we kept **TK-COF-5** crystals in *o*-DCB at room temperature for at least 47 h ("**Crystals-A1**"). Then, we acquired a PXRD pattern from **Crystals-A1** in *o*-DCB as shown in Fig. S11 and labelled with "**Crystals-A1**, as measured."

This pattern was obtained taking the average of nine scans, each of which was conducted using a slow scan rate (0.2° min^{-1}) and limiting the scan range to the low-angle region of interest to attain a sufficiently high signal-to-noise (S/N) ratio; the S/N ratio of PXRD patterns acquired in *o*-DCB is low because *o*-DCB strongly absorbs X-rays, as indicated by the large solvent background. Then, we subtracted the background to obtain the pattern labelled with "**Crystals-A1**, background removed" in Fig. S11, which did not show the ≈

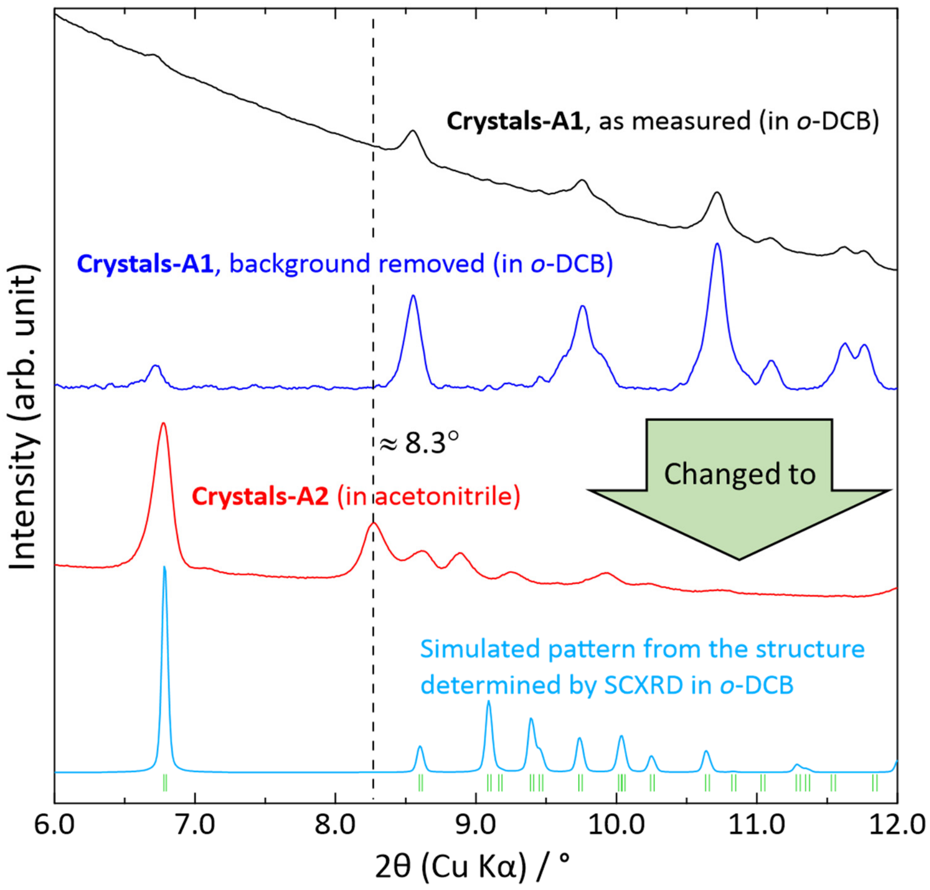

Supplementary Figure S11. The PXRD patterns acquired from **TK-COF-5** crystals in the Series A experiments and the simulated pattern generated from the crystal structure determined by the SCXRD analysis.

8.3° peak; therefore, our data of SCXRD and PXRD acquired in *o*-DCB are consistent regarding the absence of the ≈ 8.3° peak.

Subsequently, we replaced the solvent of **Crystals-A1** with acetonitrile and then kept the sample at room temperature for at least 47 h ("**Crystals-A2**"). This PXRD pattern exhibited the ≈ 8.3° peak, confirming the results shown in Fig. S10 above. These results indicate that this peak appears when **TK-COF-5** interacts with acetonitrile molecules.

Furthermore, we carried out the Series B experiments as follows. First, we kept **TK-COF-5** in acetonitrile at room temperature for at least 47 h ("**Crystals-B1**"). Then, we acquired the PXRD pattern in acetonitrile, which showed the ≈ 8.3° peak as expected (Fig. S12).

Subsequently, we replaced the solvent of **Crystals-B1** with toluene and then kept the sample at room temperature for at least 47 h ("**Crystals-B2**"). The pattern did not show the ≈ 8.3° peak. Finally, we replaced the solvent of **Crystals-B2** again with acetonitrile and then kept the sample at room temperature for at least 47 h ("**Crystals-B3**"). The ≈ 8.3° peak emerged again in the PXRD pattern (Fig. S12).

From these experimental results, we conclude that the ≈ 8.3° peak is originated from some conformation of the framework that reproducibly and specifically appears only when **TK-COF-5** interacts with acetonitrile molecules. However, because we have been unable to obtain high-quality SCXRD data in acetonitrile, the detailed structural cause of the peak found at $2\theta \approx 8.3°$ in acetonitrile has not been determined.

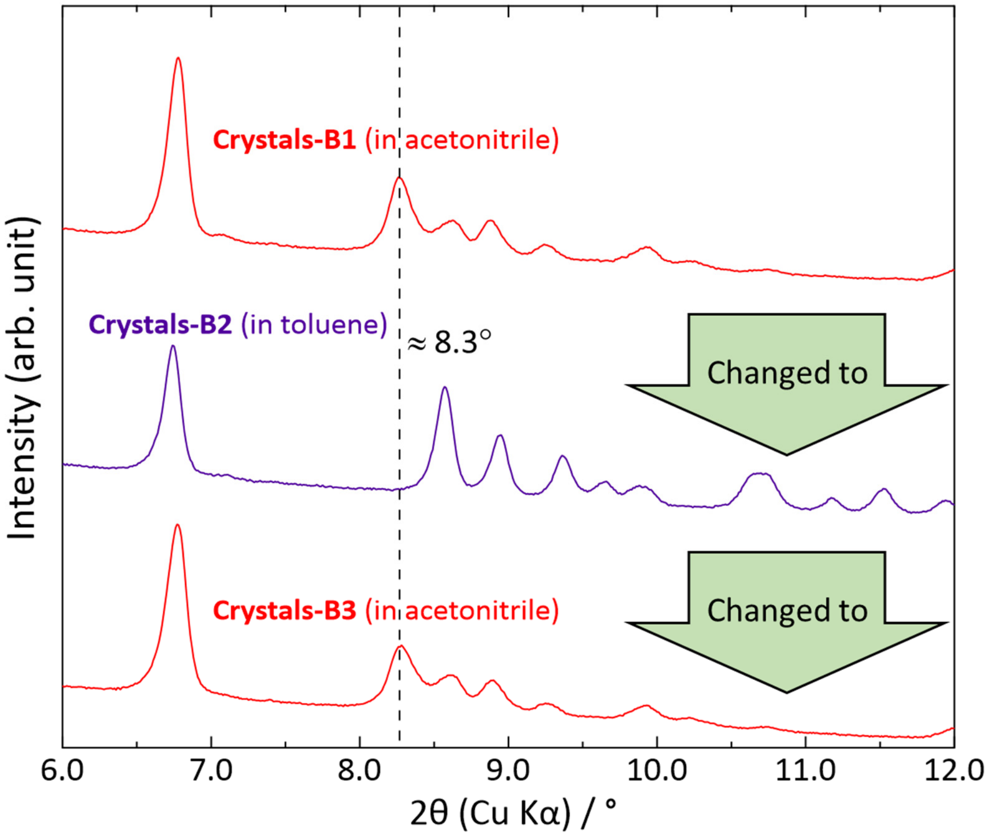

Supplementary Figure S12. The PXRD patterns acquired from **TK-COF-5** crystals in the Series B experiments.

S2.9 Determination of crystal face indexes by SCXRD measurements

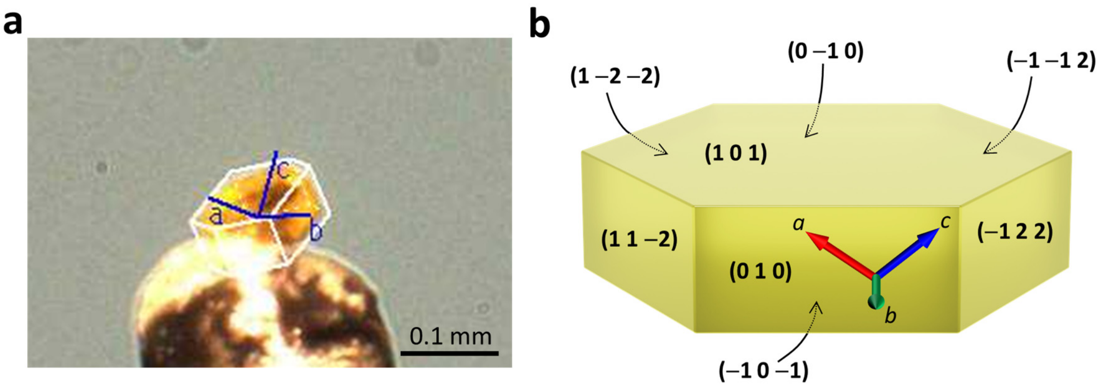

Supplementary Figure S13. a, Photograph of a **TK-COF-4** crystal taken by a camera equipped in the X-ray diffractometer (*XtaLAB Synergy-DW*, *Rigaku*) with the crystal axes and shape outline displayed by *CrysAlisPro*® software. **b**, The crystal face indexes and axes determined for a **TK-COF-4** crystal.

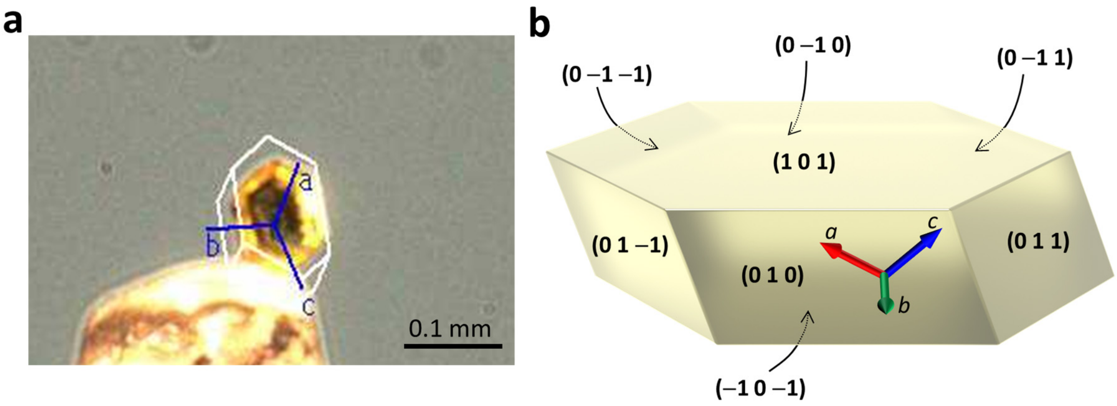

Supplementary Figure S14. a, Photograph of a **TK-COF-5** crystal taken by a camera equipped in the X-ray diffractometer (*XtaLAB Synergy-DW*, *Rigaku*) with the crystal axes and shape outline displayed by *CrysAlisPro*® software. **b**, The crystal face indexes and axes determined for a **TK-COF-5** crystal.

S2.10 Framework densities of TK-COF-4/-5 and hypothetical 3D-COFs with bor and ctn topology

We calculated the densities of **TK-COF-4/-5** and those of hypothetical COFs that have **bor** and **ctn** topology constructed by connecting **TFPT/TFPB** and **TAM** using Materials Studio® software. We used the COMPASS III force field for their geometrical optimizations. We also calculated the densities of **TK-COF-4/-5** from the structures determined by SCXRD measurements. These results are compared in Table S5. The densities of **TK-COF-4** and **-5** (*ca*. 0.7–1 g cm^{-3}) are much higher than those of the hypothetical COFs (< 0.15 g cm^{-3}). Note that the previously reported 3D-COFs with **bor** and **ctn** topology actually had low densities (see the Results and Discussion section in the main text).

Table S5. Comparison of the density of **TK-COF-4/-5** with those of hypothetical COFs with **bor** and **ctn** topology constructed by connecting **TFPT/TFPB** and **TAM**

COF type, Space group	Density calculated from the structure determined by SCXRD [g cm^{-3}]	Density of the model after geometrical optimization by Materials Studio® [g cm^{-3}]
**TK-COF-4**, *P*2$_1$/*n* (No. 14)	0.741†	0.685
Hypothetical **bor-**topological COF made from **TFPT** and **TAM**, *P*23 (No. 195)	—	0.124
Hypothetical **ctn-**topological COF made from **TFPT** and **TAM**, *I*–43*d* (No. 220)	—	0.137
**TK-COF-5**, *P*2$_1$/*n* (No. 14)	0.684†	0.744
**TK-COF-5_dried**, *P*2$_1$/*n* (No. 14)	1.03	1.09
Hypothetical **bor-**topological COF made from **TFPB** and **TAM**, *P*23 (No. 195)	—	0.119
Hypothetical **ctn-**topological COF made from **TFPB** and **TAM**, *I*–43*d* (No. 220)	—	0.130

† Solvent molecules were excluded in the density calculations.

S2.11 Area densities of primary amines of TK-COF-4/-5 and other COFs

We calculated the area densities of primary amines in **TK-COF-4** and **-5** per layer from their structures determined from the SCXRD data analyses. We also calculated the area densities of primary amines in previous COFs per layer from their reported CIF or atomic coordinates. We compared these area densities in Table S6. See the Results and Discussion section in the main text for discussion.

Supplementary Table S6. Comparison of the area density of primary amines in **TK-COF-4/-5** with those in other COFs

COF name	Type	Area density of primary amines [$\times 10^5$ μm^{-2}]	Ref.
**TK-COF-4**	Unreacted primary amines remaining after the synthesis of COFs	4.9	This work
**TK-COF-5**		4.6	
**PT-/PY-COF**		1.6†	S6
**NH_2-Th-Tz COF**		1.7†	S7
**COF-609**	Primary amines appended to 2D-COFs by post-synthetic modification	5.3†	S8
**Me_3TFB-$(NH_2)_2BD$**		8.0†	S9
**COF-284-NH_2**		15†	S10
**COF-285-NH_2**		9.2†	
**COF-999**		5.1†	S11

† Calculated by us from the reported COF structure using Materials Studio® software.

S2.12 Effect of solvent removal on crystal structure

The PXRD patterns of the dried crystals shown in Fig. S4 are largely different from those of the crystals suspended in acetonitrile (Fig. 1g in the main text). To investigate the effect of the removal of solvent on the structure, we carried out the following investigations.

After repeating trials many times, we succeeded in an acquisition of SCXRD data with sufficiently high quality from a dried crystal of **TK-COF-5**, which we denote by **TK-COF-5_dried**. The data were analyzed following the procedure similar to that used for **TK-COF-4/-5** above, from which the asymmetric unit has been determined as shown in Fig. S15. The crystallographic data are summarized in Supplementary Table S7.

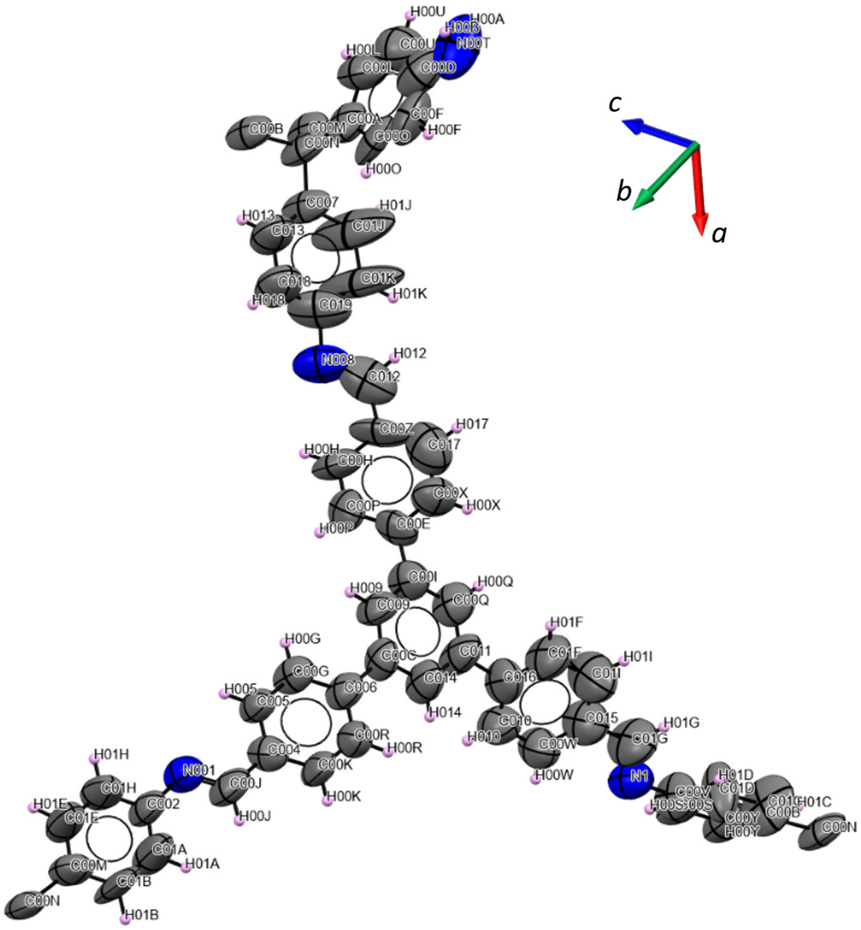

Supplementary Figure S15. Asymmetric unit of the structure of **TK-COF-5_dried** determined from SCXRD data and structural analysis. Thermal ellipsoids are drawn with 50% probability.

Supplementary Table S7. Crystallographic data for **TK-COF-5_dried**

**TK-COF-5_dried**	
Chemical composition of COF	$C_{52}H_{36}N_4$
Formula mass	716.85
Crystal system	Monoclinic
Space group	$P2_1/n$
a, Å	8.1731(19)
b, Å	39.015(7)
c, Å	14.575(5)
β, °	97.20(3)
V, Å^3	4611(2)
d, g cm^{-3}	1.033
μ, mm^{-1}	0.468
Z	4
Reflections collected	27625
Independent reflections	9037
Observed reflections	9037
Restraints	120
Parameters	506
2θ range for data collection, °	4.53 to 169.092
Index ranges	$-10 \le h \le 7$, $-47 \le k \le 48$, $-18 \le l \le 18$
R_{int}	0.2134
R_σ	0.1703
Goodness-of-fit on F^2	1.097
Final R indexes $(I \ge 2\sigma(I))$	$R_1 = 0.1805$, w$R_2 = 0.4404$
Final R indexes [all data]	$R_1 = 0.4036$, w$R_2 = 0.5553$
$\Delta\rho_{max}$, e Å^{-3}	0.32
$\Delta\rho_{min}$, e Å^{-3}	–0.24
Crystal size, mm^3	0.1 × 0.06 × 0.04
Radiation, Å	Cu Kα (λ = 1.54184)
Temperature, K	93
CCDC number	2383526

The simulated PXRD pattern reconstructed from the structure of **TK-COF-5_dried** determined from the SCXRD analysis is compared with the measured PXRD pattern in Fig. S16 below, showing a satisfactory agreement between them.

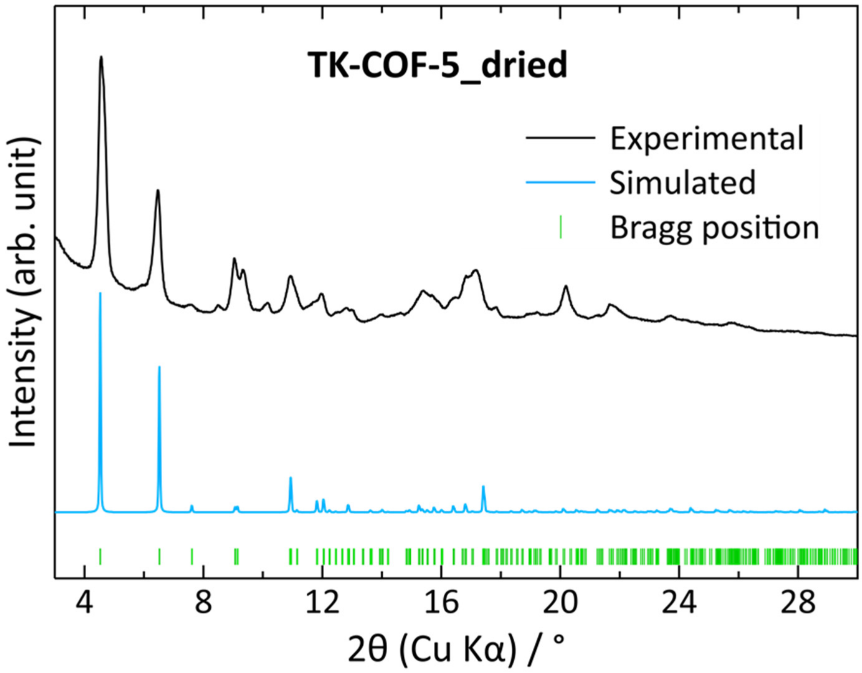

Supplementary Figure S16. Comparison of the PXRD pattern from **TK-COF-5_dried** crystals in acetonitrile with the simulated pattern generated from the crystal structure determined by the SCXRD analysis.

The framework structures of **TK-COF-5** (in *o*-DCB) and **TK-COF-5_dried** are compared graphically in Fig. S17. These structures are qualitatively the same. However, as compared on the bottom of Fig. S17 (**TK-COF-5** *vs*. **TK-COF-5_dried**), the framework of **TK-COF-5_dried** is slightly deformed from that of **TK-COF-5** (in *o*-DCB). Therefore, this difference in the structure is found to be the reason for the difference in their PXRD patterns.

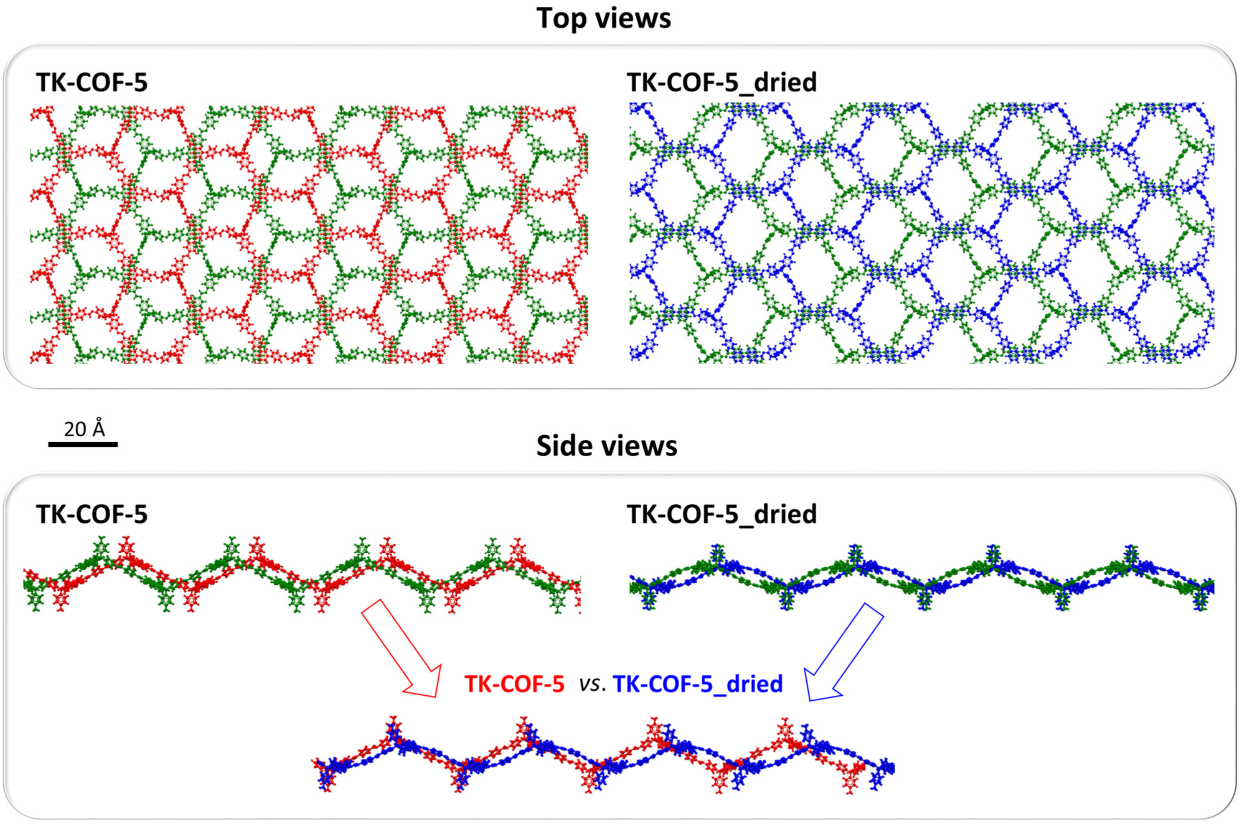

Supplementary Figure S17. Comparison of the structure of **TK-COF-5**, which was determined by the SCXRD data obtained in *o*-DCB (Fig. 2a in the main text, Fig. S9; see also Methods in the main text), and that of **TK-COF-5_dried** (Fig. S15). The frameworks in green color represent an interpenetrated framework to the unit framework of **TK-COF-5** (red) and that of **TK-COF-5_dried** (blue).

S2.13 Assessment of thermal stability

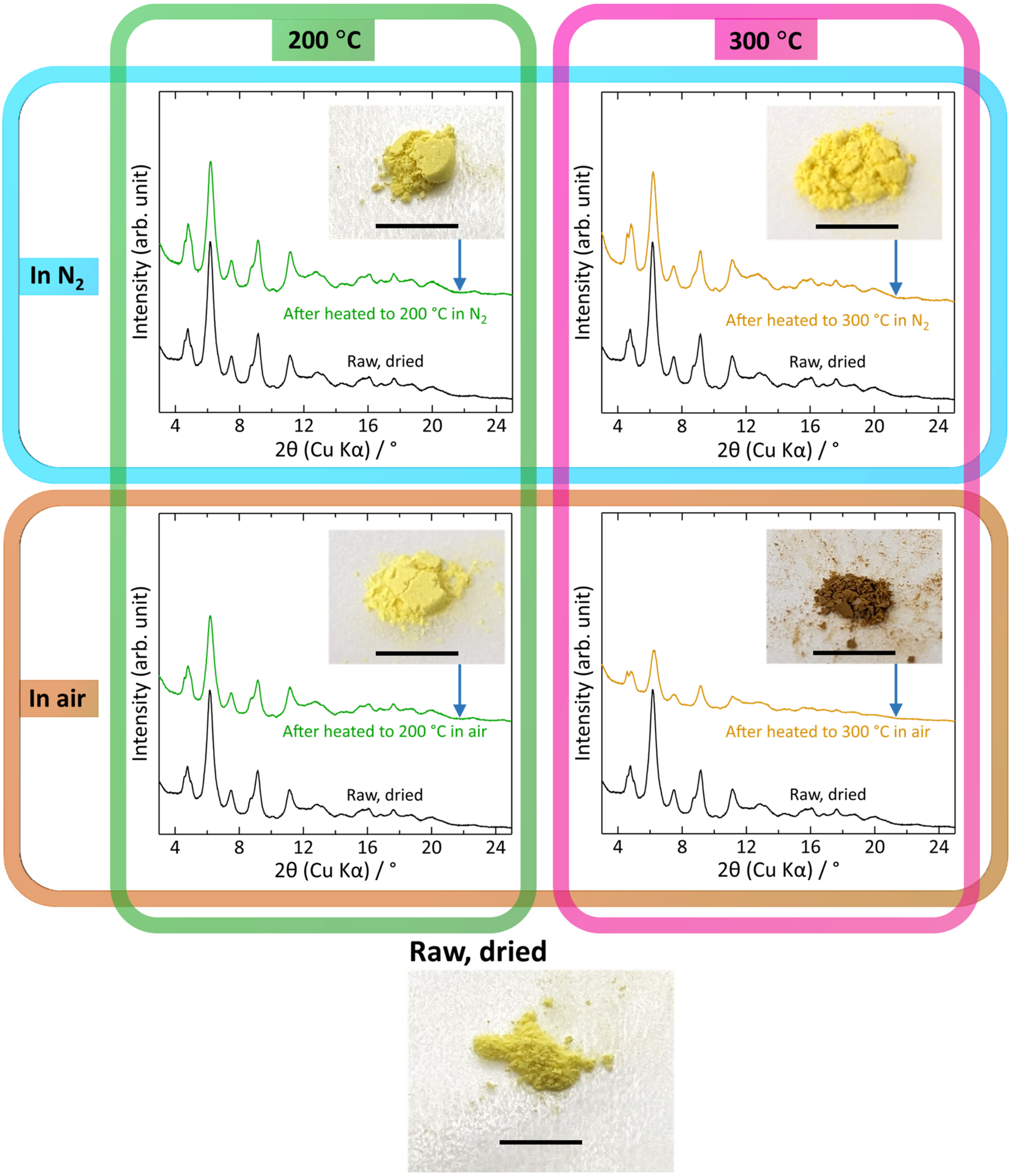

Supplementary Figure S18. The PXRD patterns and photographs of **TK-COF-4** after heating to 200 (left) or 300 °C (right) in nitrogen (top) or air (bottom). Scale bars are 5 mm.

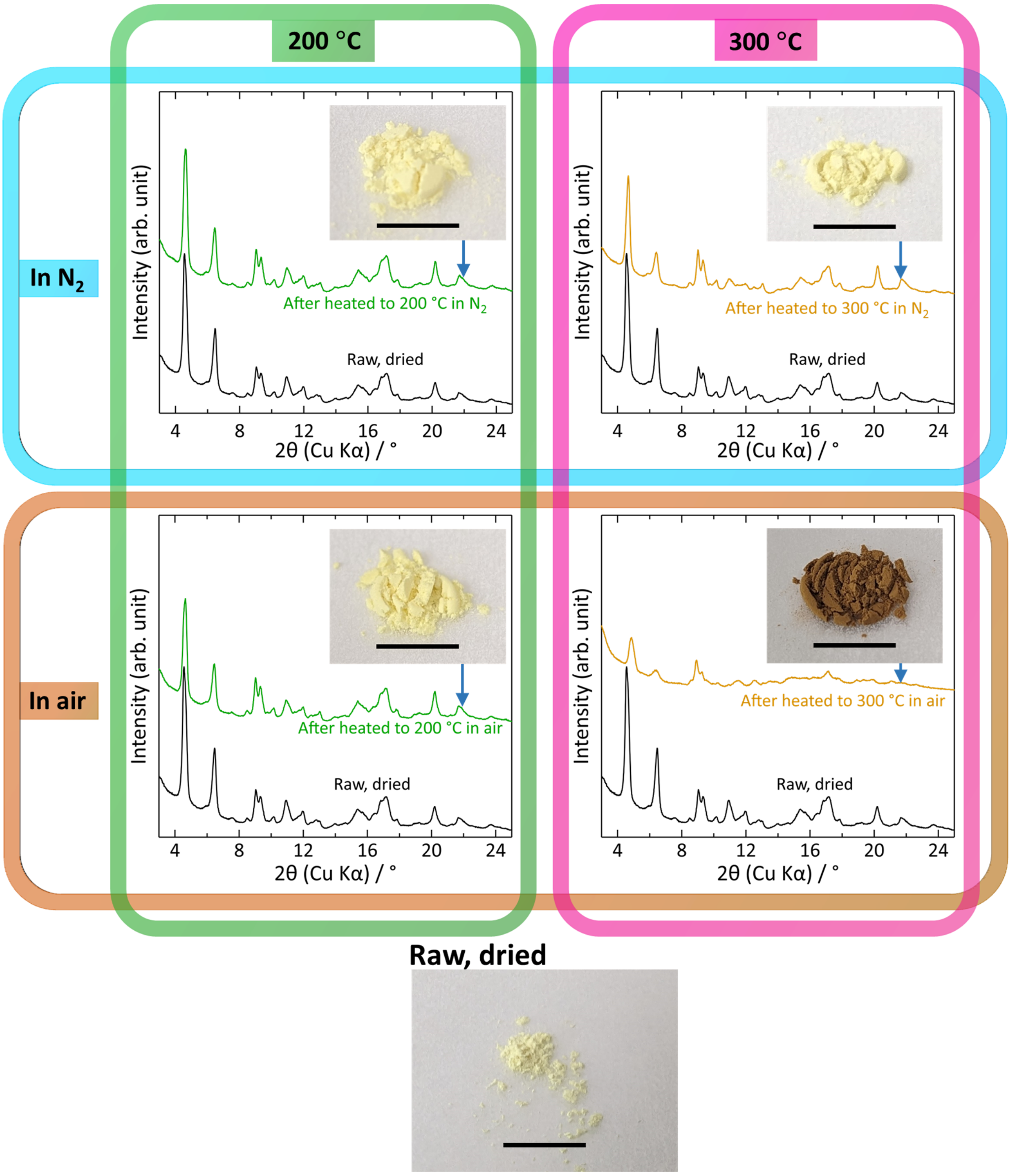

Supplementary Figure S19. The PXRD patterns and photographs of **TK-COF-5** after heating to 200 (left) or 300 °C (right) in nitrogen (top) or air (bottom). Scale bars are 5 mm.

Oxidative degradation of amines must be considered when **TK-COF-4/-5** are to be used as sorbent for CO_2 capture, because materials are often held at elevated temperature during the regeneration process. Previously, Srikanth et al[S12]. investigated oxidative degradation of silica-supported amine sorbents and reported that their CO_2 capture capacities decreased drastically after thermal treatment in air for 12 h at 100 °C, which is the typical regeneration temperature of amine sorbents.

We compared CO_2 adsorption isotherms at 273 K before and after we exposed **TK-COF-4** to air at 100 °C for 12 h using a digitally temperature-controlled bath. As shown by Fig. S20, the invariant CO_2 capacity from this treatment indicates high thermal stability against amine oxidation in the present COF. Furthermore, as shown in Fig. S21, the PXRD pattern and FT-IR spectrum after this thermal treatment were the same as those acquired before the

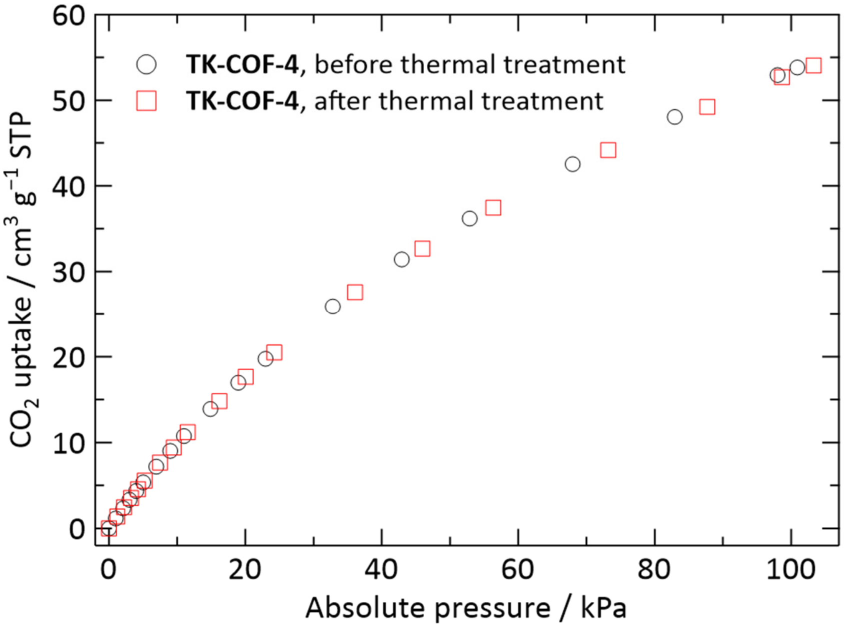

Supplementary Figure S20. Comparison of CO_2 adsorption isotherms measured for **TK-COF-4** at 273 K before and after the thermal treatment in air at 100 °C for 12 h.

treatment, supporting the high stability. Such a high stability against oxidation may have arisen from the low basicity of aromatic amines and is considered advantageous in a sorbent for CO_2 capture.

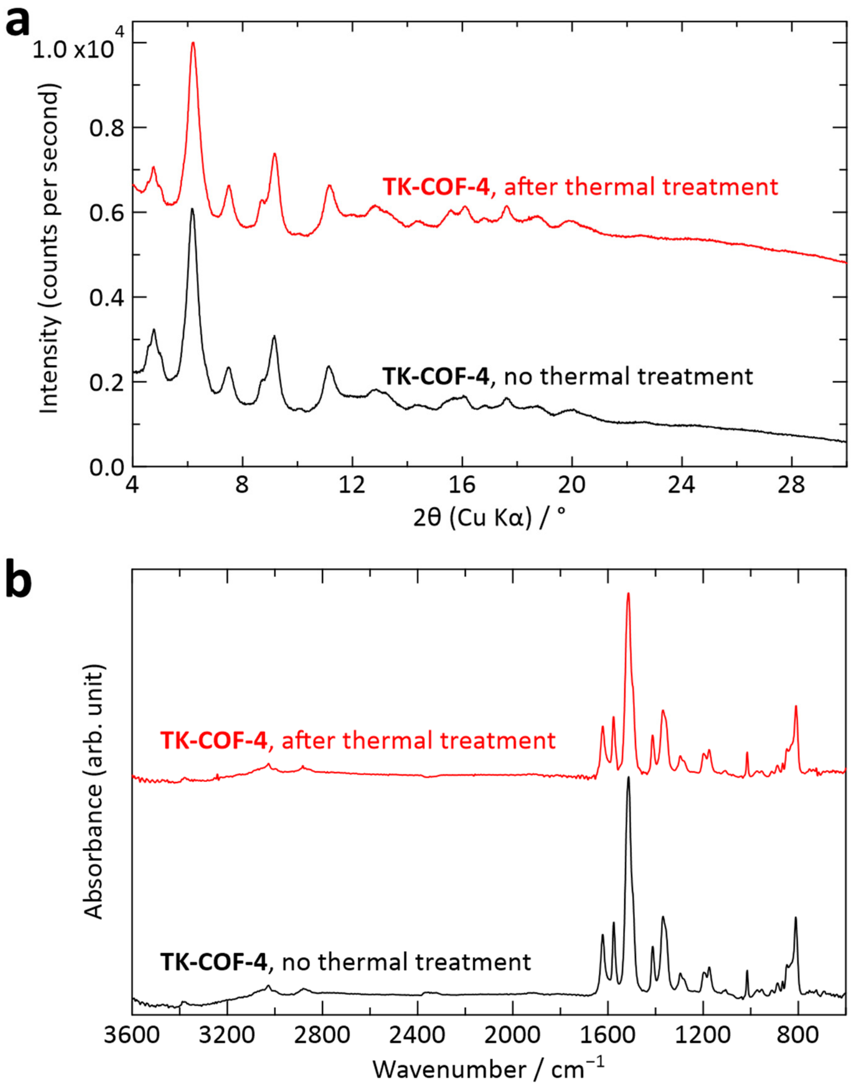

Supplementary Figure S21. a, PXRD pattern and **b**, FT-IR spectrum of **TK-COF-4** before and after the thermal treatment in air at 100 °C for 12 h.

S2.14 Results of gas adsorption measurements and analyses

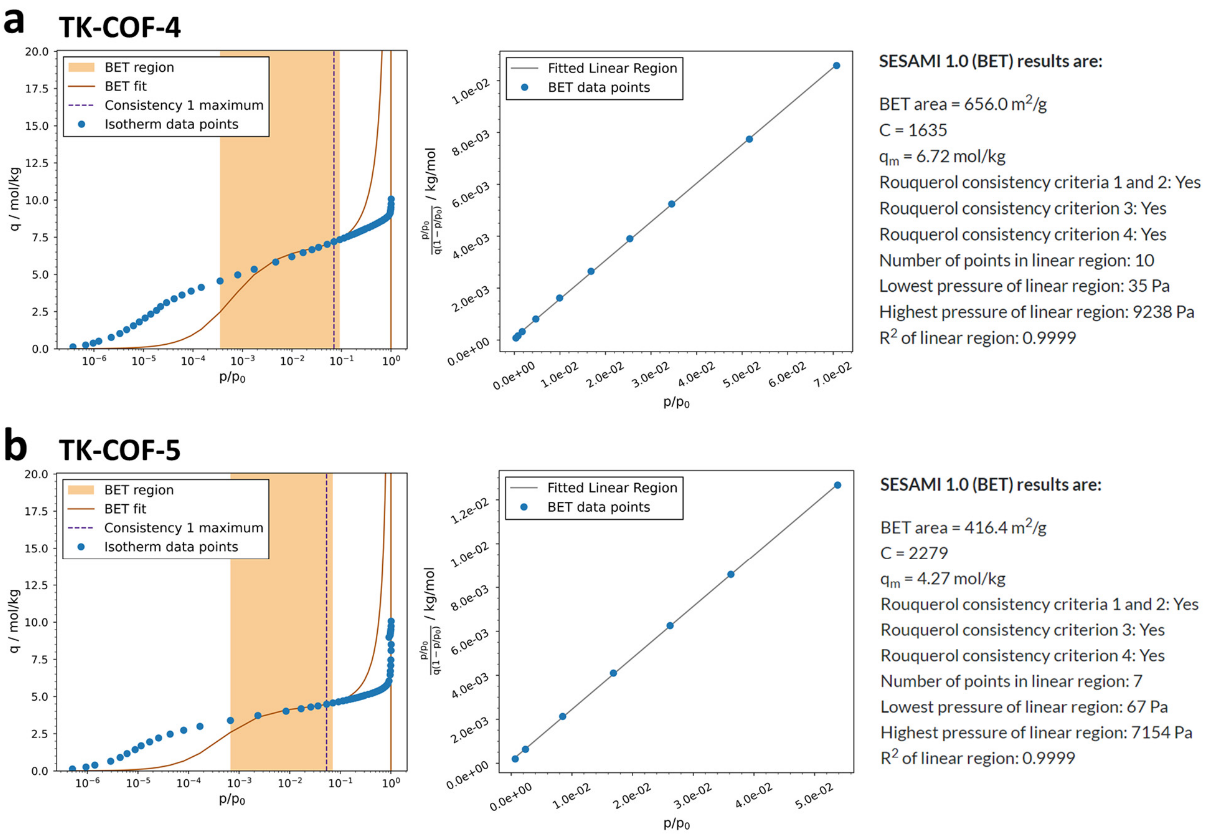

Supplementary Figure S22. Results of BET surface area analyses for **a**, **TK-COF-4**, **b**, **TK-COF-5** on the N_2 adsorption isotherms at 77 K shown in Fig. 3c in the main text using the SESAMI 1® algorithm in the SESAMI® web interface. The calculated BET surface areas are shown in the first line of the output from SESAMI 1 on the right of each panel.

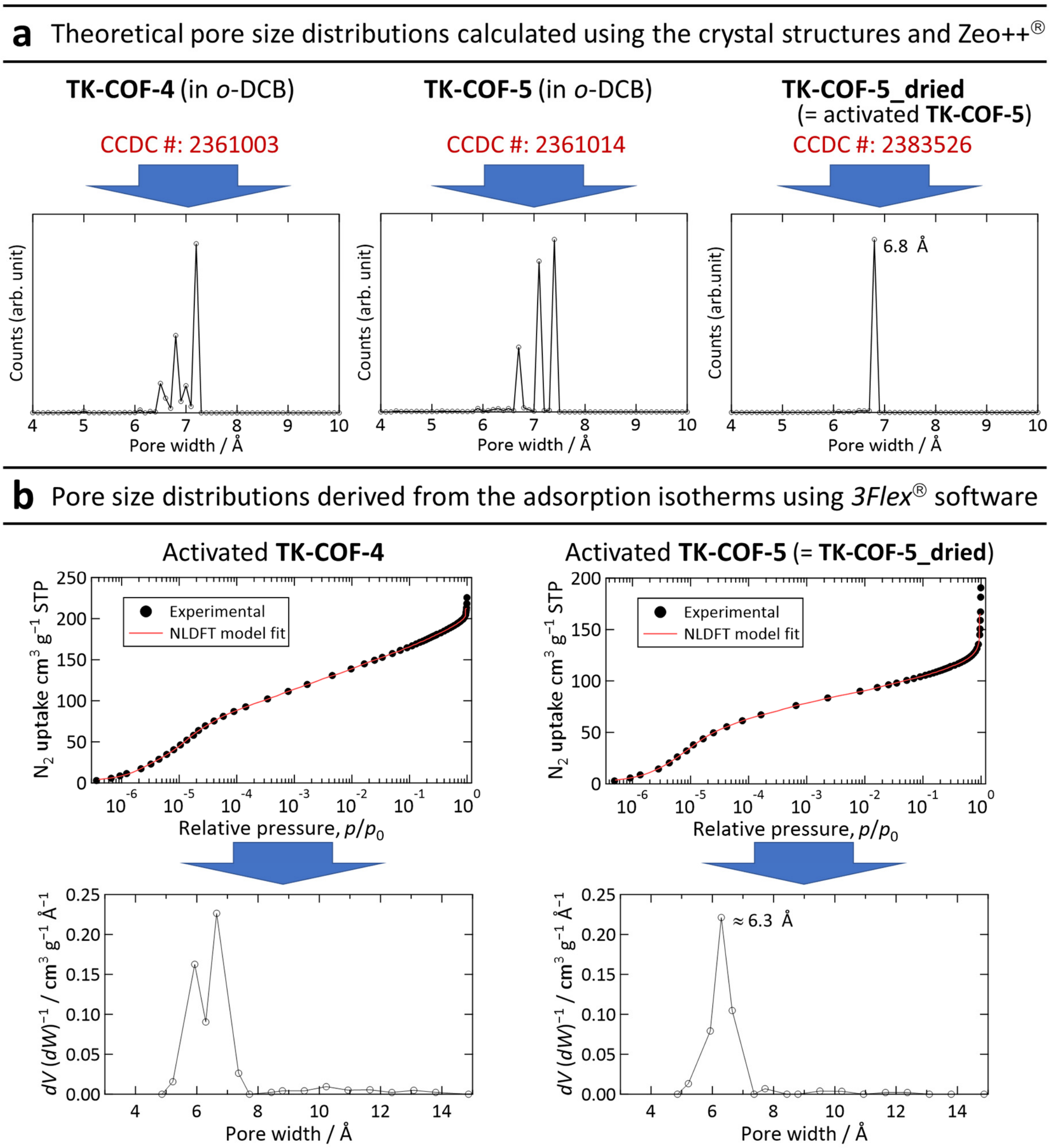

Supplementary Figure S23. **a**, Theoretical pore size distributions calculated from the crystal structure data of **TK-COF-4** (in *o*-DCB, CCDC#: 2361003), **TK-COF-5** (in *o*-DCB, CCDC#: 2361014), and **TK-COF-5_dried** (activated, CCDC#: 2383526) using Zeo++® software version 0.2.0 with a 1.67-Å radius probe N_2 molecule, the high-accuracy flag, and 100,000 Monte Carlo samples per unit cell. **b**, Pore size distributions derived by fitting the NLDFT model to the measured N_2 adsorption isotherms at 77 K using *3Flex* software with the model of "N2–Tarazona NLDFT (Esf: 30.0 K)" and geometry of "Cylinder."

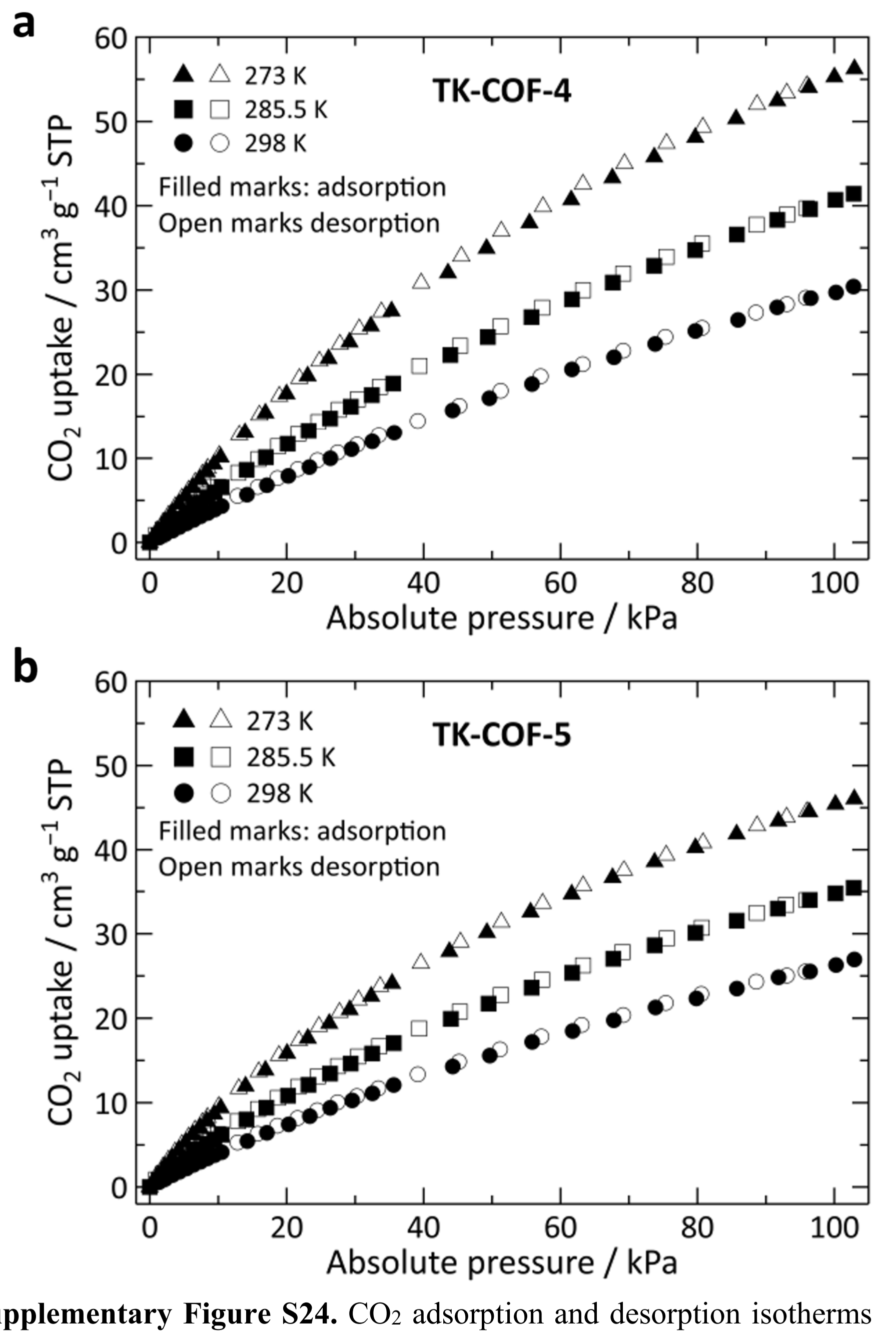

Supplementary Figure S24. CO_2 adsorption and desorption isotherms for **a**, **TK-COF-4**, **b**, **TK-COF-5** acquired at 273, 285.5, and 298 K

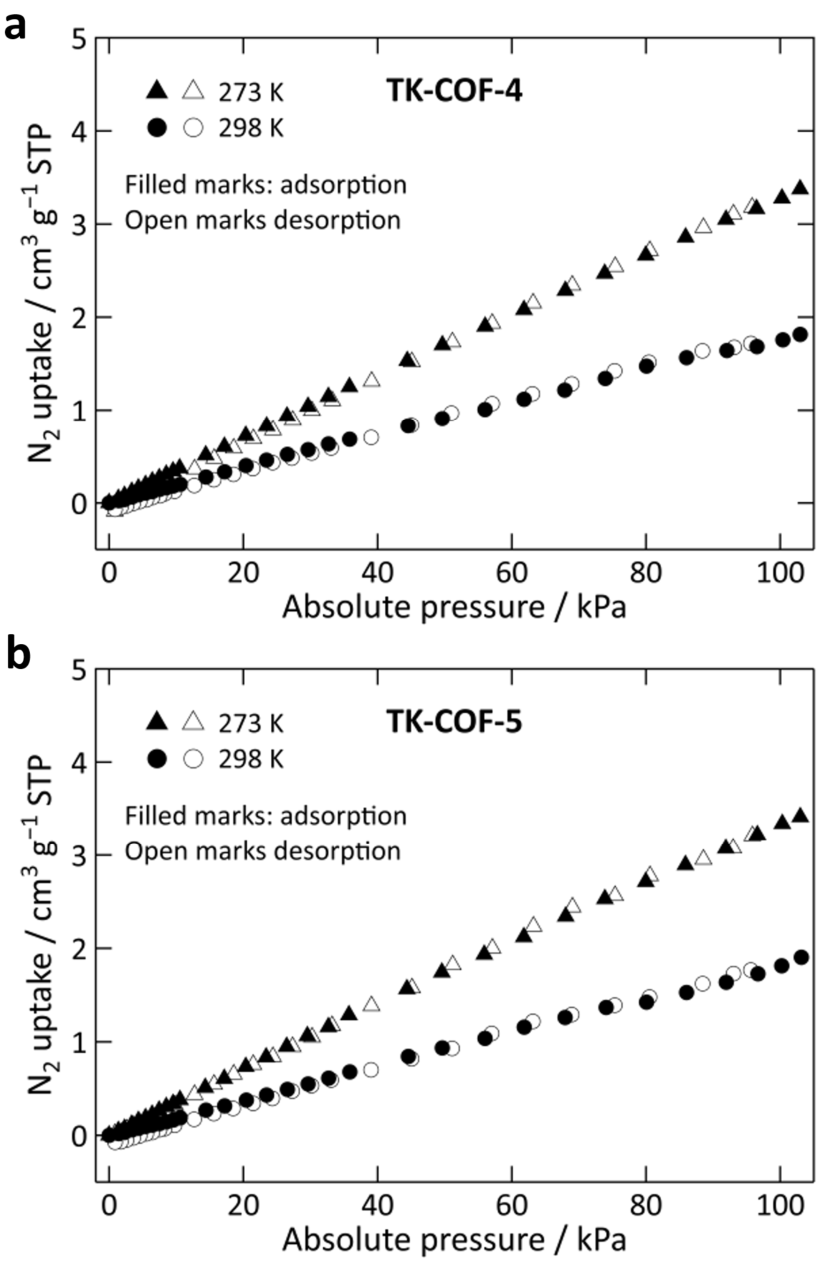

Supplementary Figure S25. N_2 adsorption and desorption isotherms for **a**, **TK-COF-4**, **b**, **TK-COF-5** acquired at 273 and 298 K

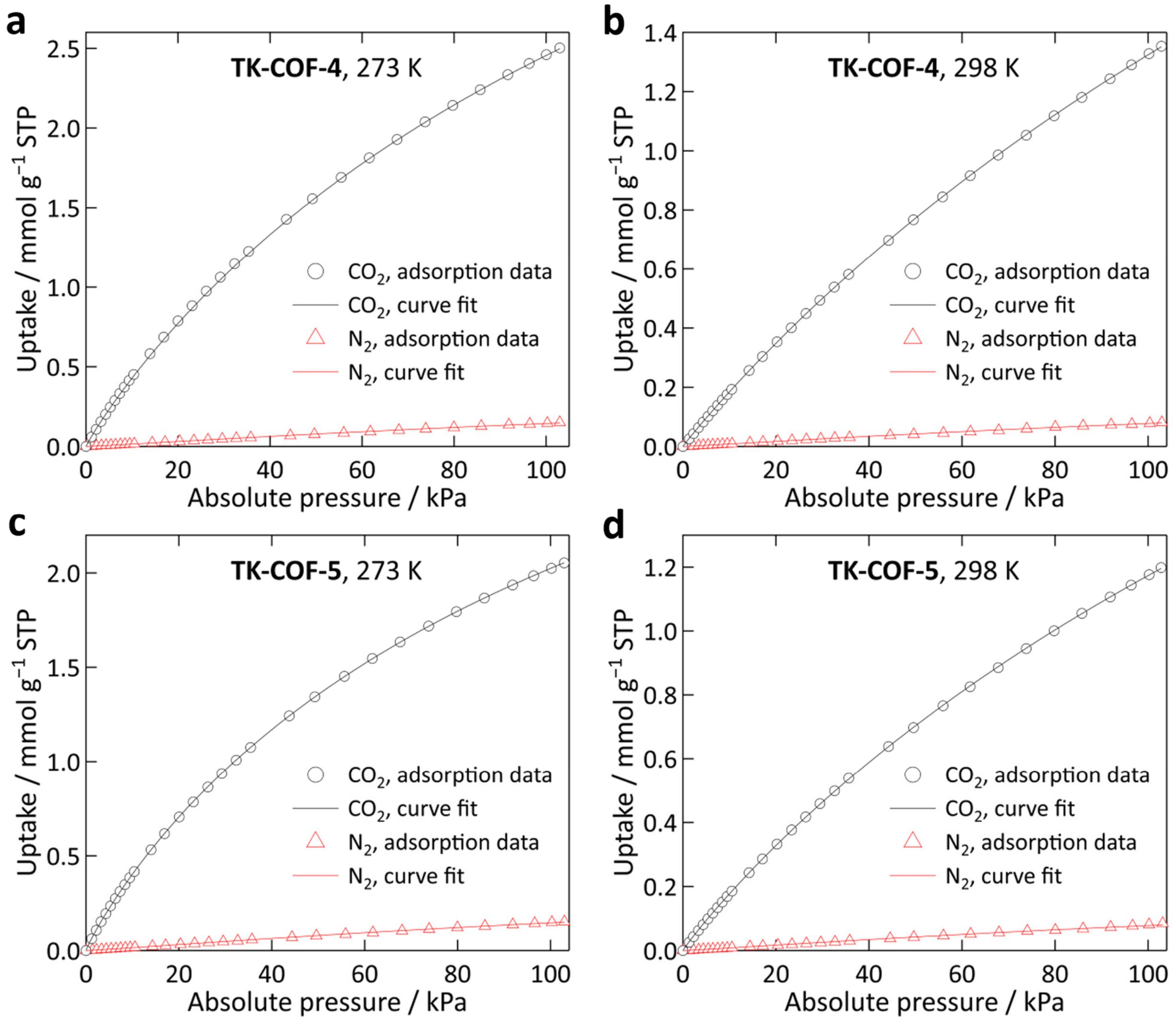

Supplementary Figure S26. Curve fits to the adsorption data by the Langmuir–Freundlich model (black: CO_2, red: N_2). **a** and **b**, **TK-COF-4** at 273 and 298 K, respectively. **c** and **d**, **TK-COF-5** at 273 and 298 K, respectively. The parameters used in the fittings are summarized in Table S8.

Supplementary Table S8. Summary of the parameters obtained from the curve fits by the Langmuir–Freundlich model in Fig. S26.

COF name	Adsorbed gas (temperature)	*a* [mmol g^{-1}]	*b* [MPa^{-c}]	*c*
**TK-COF-4**	CO_2 (273 K)	6.556	8.1589	0.93278
	N_2 (273 K)	0.5308	2.2854	1.1053
	CO_2 (298 K)	5.2691	3.9187	0.96638
	N_2 (298 K)	0.28616	2.3453	1.1001
**TK-COF-5**	CO_2 (273 K)	4.5602	11.797	0.91511
	N_2 (273 K)	0.5353	2.2546	1.1112
	CO_2 (298 K)	4.2414	4.952	0.94393
	N_2 (298 K)	0.27603	2.3004	1.1163

S2.15 Advantage in CO_2 adsorption rate of the highly crystalline material over the reference material with lower crystallinity

TK-COF-4 and **-5** have high crystallinity and ordered pores even after the solvent removal, as demonstrated above. The high crystallinity of the materials and highly ordered pores are considered to be advantageous in the dynamic rate of CO_2 adsorption against the materials that have the same chemical composition but lower crystallinity and less-ordered pores. To investigate whether such an expected advantage exists, we carried out the following experiments in which we compared crystalline **TK-COF-4**, which had better CO_2 adsorption performance than **TK-COF-5** (see the main text), and the amorphous version of **TK-COF-4**, which we named **TK-COF-4-LC**, where "LC" stands for "low crystallinity."

TK-COF-4-LC was created by drastically decreasing the amount of modulator used during its growth. Specifically, we used the modified Condition II (see Table 1 in the main text) with a reduced aniline amount of 3 equiv., which is approximately 1/10 of the original amount of aniline used to generate crystalline **TK-COF-4**. The optical microscopy (Fig. S27a) and SEM (Fig. S27b) images indicate the low crystallinity of **TK-COF-4-LC**. Furthermore, the comparison of the PXRD patterns of **TK-COF-4-LC** and **TK-COF-4** (Fig. S28) reveals that the former is almost amorphous and hence has a much lower crystallinity than the latter, as we intended.

Notably, the FT-IR spectra of **TK-COF-4-LC** and **TK-COF-4** were identical (Fig. S29), suggesting that their chemical compositions are the same. These results demonstrate that **TK-COF-4-LC** is a suitable reference material to study the aforementioned aim—to investigate

whether the high crystallinity of **TK-COF-4** is an advantage in the dynamic CO_2 adsorption rate.

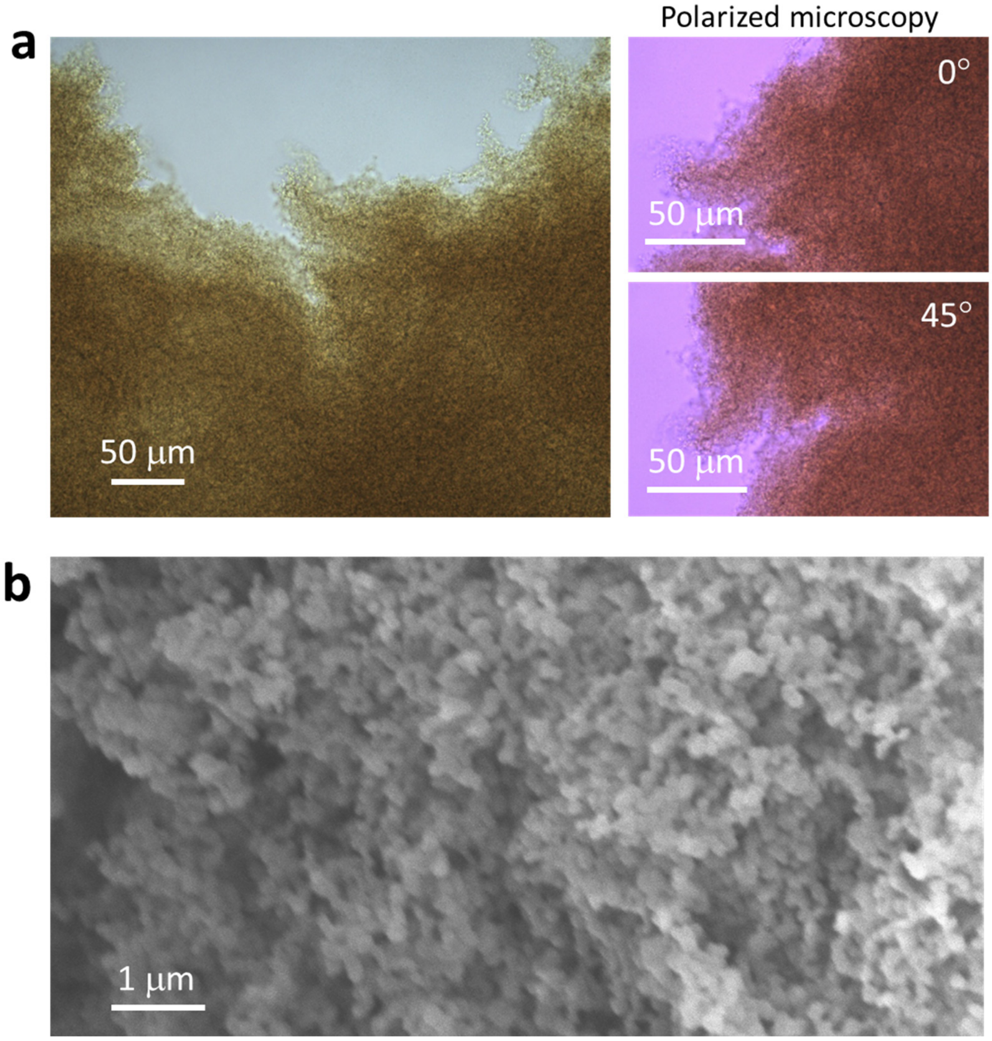

Supplementary Figure S27. Microscope images of **TK-COF-4-LC**. **a**, Optical microscope images; **b**, SEM images.

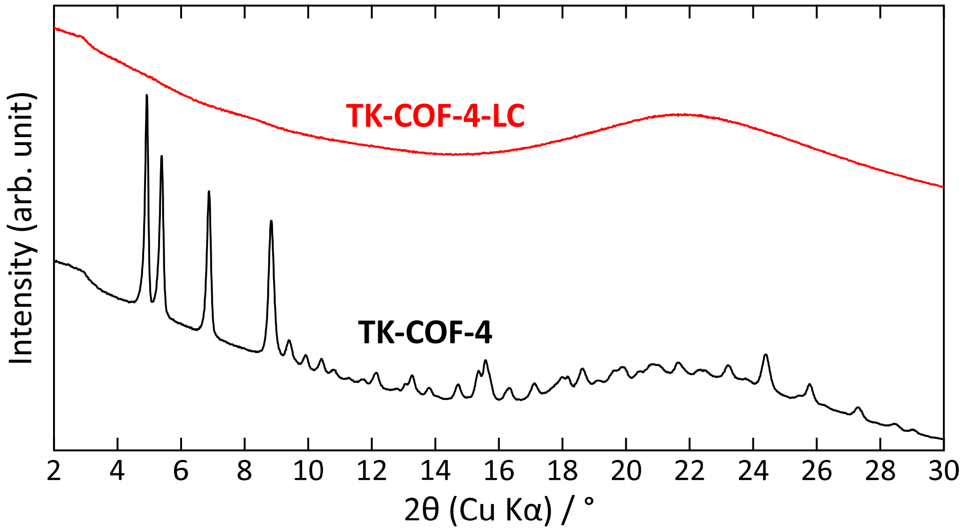

Supplementary Figure S28. Comparison of PXRD patterns of **TK-COF-4-LC** and **TK-COF-4**.

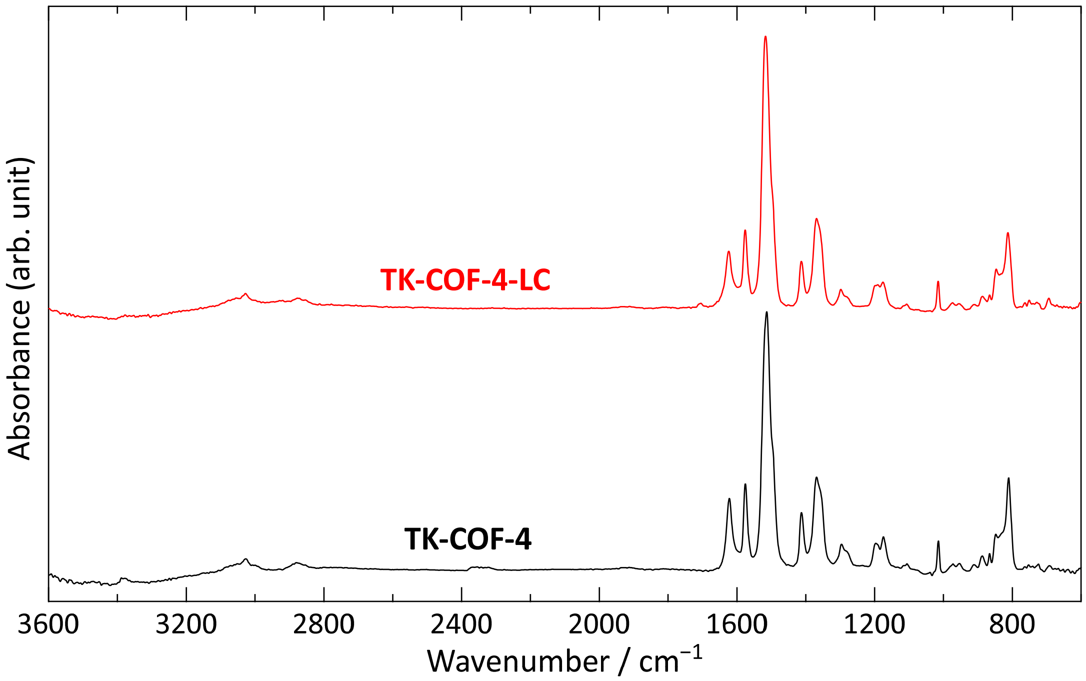

Supplementary Figure S29. Comparison of FT-IR spectra of **TK-COF-4-LC** and **TK-COF-4**.

The N_2 adsorption capacity at 77 K (Fig. S30) and CO_2 adsorption capacity at 273 K (Fig. S31) of **TK-COF-4-LC**, after the same washing and activation procedures as those taken for **TK-COF-4/-5**, were *ca*. 10 and 25% lower, respectively, than those of **TK-COF-4**. These results indicate that the equilibrium CO_2 adsorption capacity of **TK-COF-4-LC** is lower than that of **TK-COF-4** but that their adsorption capacities are still of the same order of magnitude. These properties also indicate that **TK-COF-4-LC** is a suitable reference to be compared with **TK-COF-4** for the present purpose.

We carried out dynamic CO_2 adsorption experiments using the time-resolved measurement mode of our *3Flex (Micromeritics)* gas sorption analyzer. In this mode, the temporal change of the pressure in a sample tube is recorded after a pulse-wise addition of the prescribed amount of CO_2 into the tube. The time constant of the decay of the CO_2 pressure toward a new equilibrium pressure in the sample tube, which is caused by adsorption of CO_2 by the

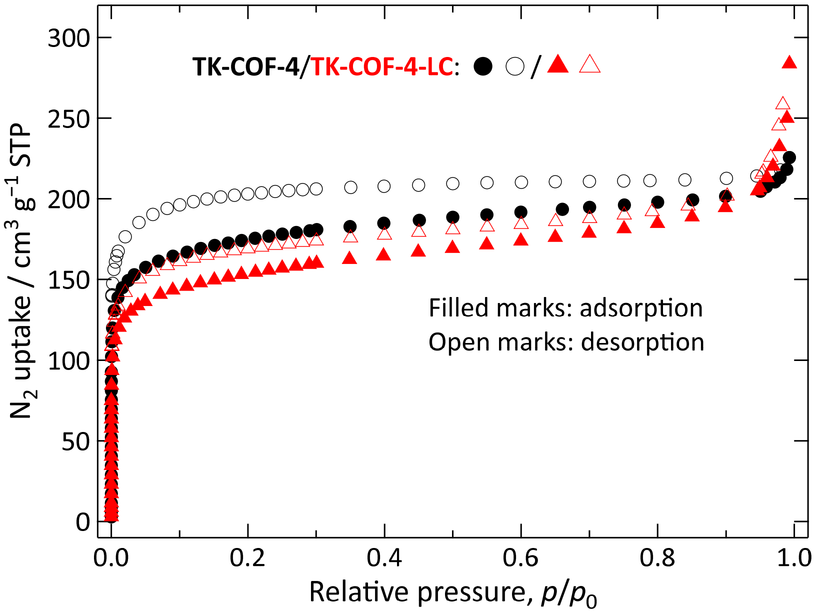

Supplementary Figure S30. Comparison of N_2 adsorption isotherms at 77 K of **TK-COF-4-LC** and **TK-COF-4**.

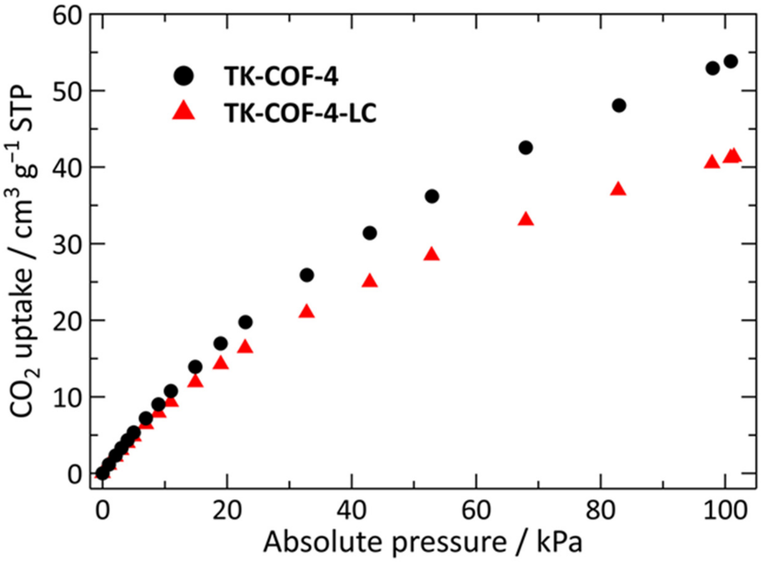

Supplementary Figure S31. Comparison of CO_2 adsorption isotherms at 273 K of **TK-COF-4-LC** and **TK-COF-4**.

sample, represents the kinetics or speed of the gas diffusion and adsorption in the adsorbent loaded in the tube. If the highly ordered structure of **TK-COF-4** is advantageous, as expected, a faster decay of the CO_2 pressure to a new equilibrium pressure would be observed in the data from this dynamic CO_2 adsorption experiment.

To conduct this experiment as reliably as possible, we loaded the same amount (95.9 ± 1.8 mg) of samples for **TK-COF-4-LC** and **TK-COF-4** in two sample glass tubes (Fig. S32). These samples were degassed under vacuum at elevated temperatures following the procedure described in Methods in the main text. The adsorption experiments were conducted at 273 K using two sample ports of *3Flex* to measure these samples simultaneously.

In our program, the CO_2 pressure started from 0 kPa and increased with stepwise additions of CO_2 gas into the sample tube until the pressure reached 101 kPa. At the beginning of each step, CO_2 was instantaneously added to the sample tube, which caused a stepwise increase

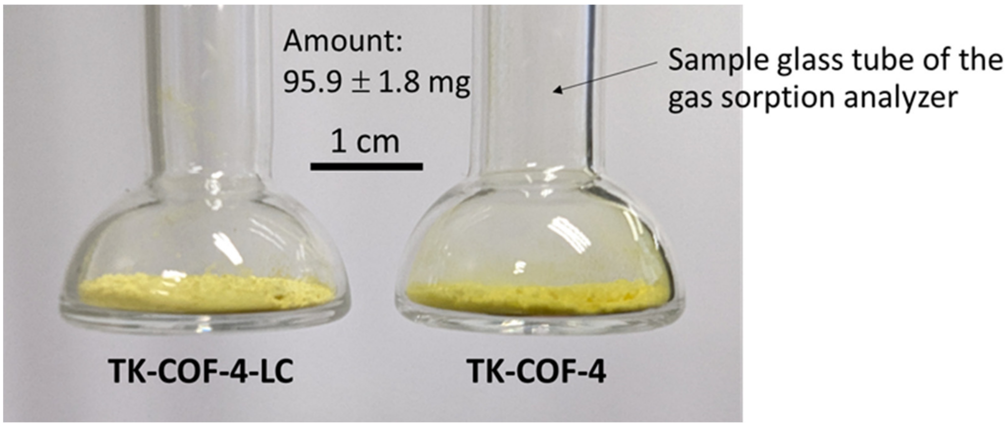

Supplementary Figure S32. Photographs of **TK-COF-4-LC** and **TK-COF-4** loaded in the sample glass tubes of the gas sorption analyzer to carry out the dynamic CO_2 adsorption experiments.

of CO_2 pressure in the tube. Beginning right after this addition, the pressure in the tube started to decay toward a new equilibrium pressure. This decay of the CO_2 pressure, caused by the transient adsorption of CO_2 by the material in the sample tube, was fit with a single exponential decay function to obtain the time constant τ for each step.

Figure S33 shows the results of the dynamic adsorption experiment for **TK-COF-4-LC** (Fig. S33a) and **TK-COF-4** (Fig. S33b), each of which consisted of a 20-step increase of CO_2 pressure from 0 to 101 kPa. In Figs. S33c and S33d, some typical pressure decays for the results of **TK-COF-4-LC** and **TK-COF-4**, respectively, are shown with the curves fitted by a single-exponential decay function and the resultant τ value.

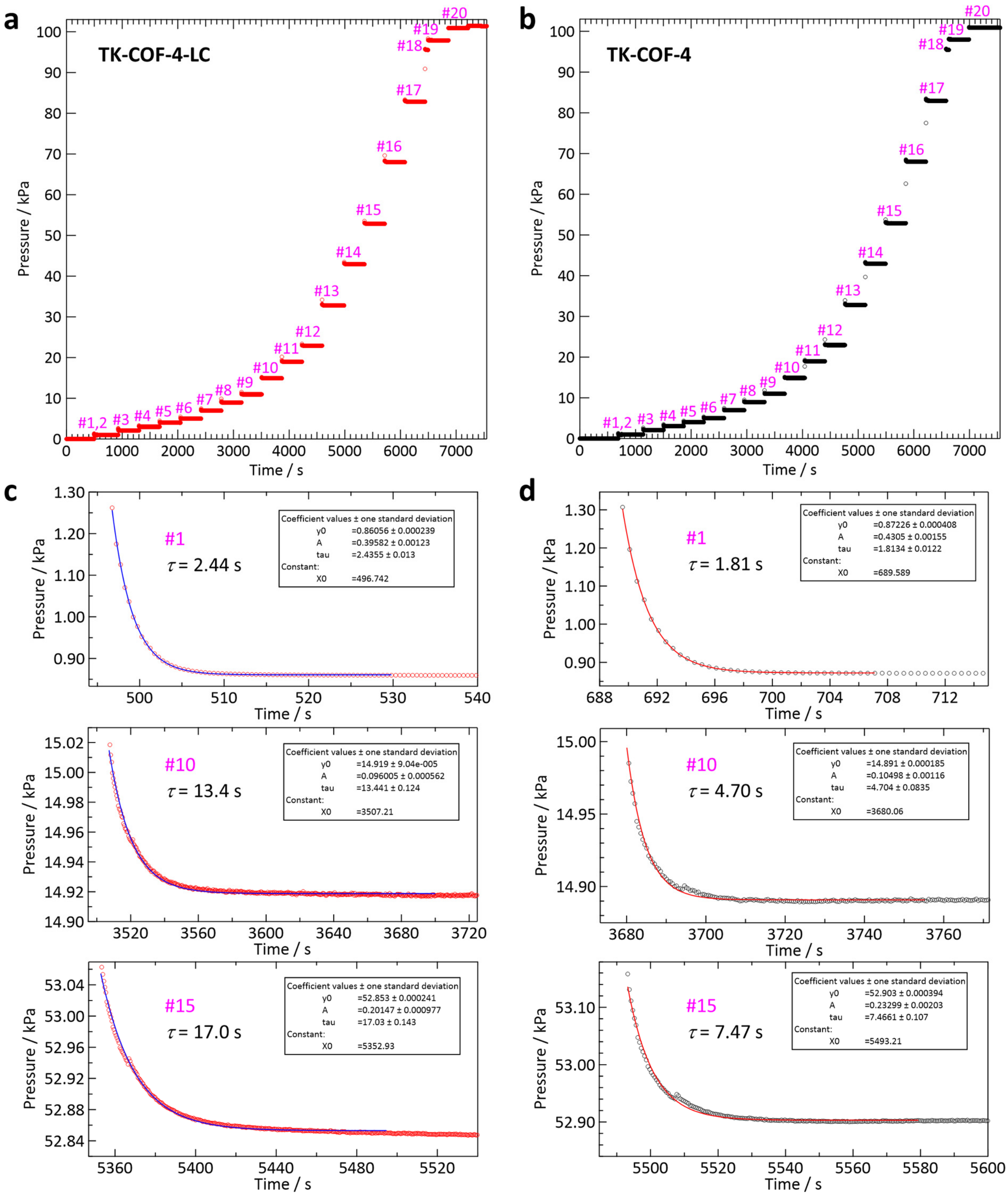

Supplementary Figure S33. (**a** and **b**) Results of the dynamic CO_2 adsorption experiments for **TK-COF-4-LC** and **TK-COF-4**, respectively. (**c** and **d**) Typical pressure decays and the curve fits by single-exponential decay functions shown for steps #1, #10, and #15 of the results for **TK-COF-4-LC** and **TK-COF-4**, respectively.

The overall results are summarized in Fig. S34. As shown in the figure, the values of τ for **TK-COF-4-LC** were approximately a factor of two larger than those of **TK-COF-4**, indicating the faster CO_2 adsorption kinetics in **TK-COF-4**, which has high crystallinity. The monotonic increase of τ with the pressure is considered to reflect the Langmuir-type (*i.e.*, site-occupation type) adsorption by these materials (*cf.* Fig. S31). The results demonstrate that, as we expected, the high crystallinity of **TK-COF-4** is advantageous to realize faster CO_2 adsorption, presumably owing to the ordered pores or gas diffusion channels present as a result of the high crystallinity.

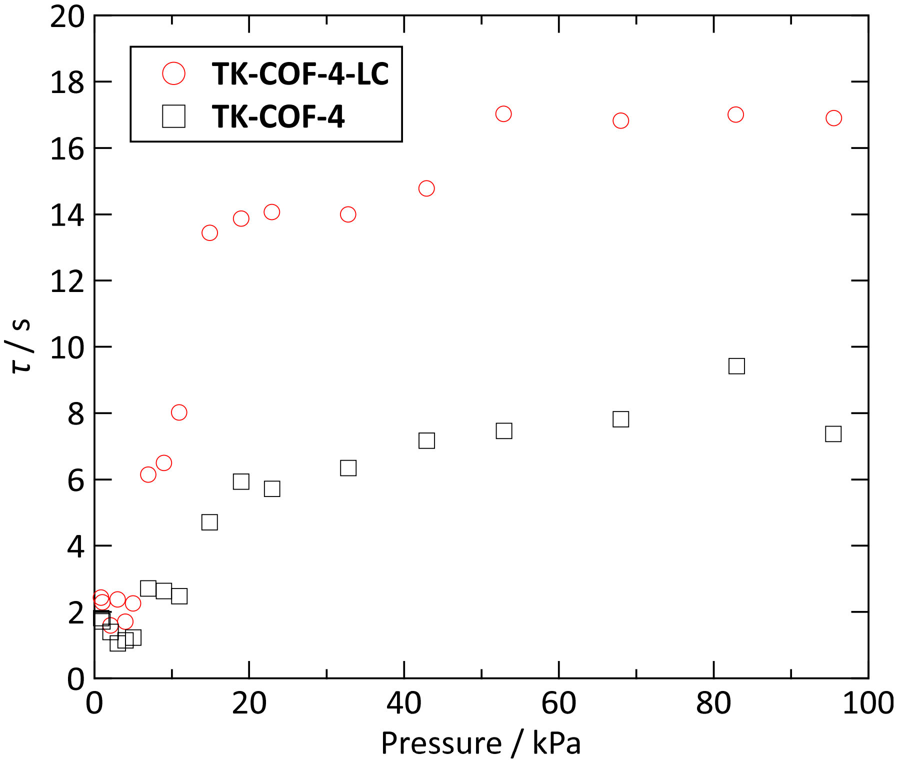

Supplementary Figure S34. Plots of the decay time constants obtained by the fits to the data for steps #1 to 18 of the dynamic CO_2 adsorption experiments in Fig. S33 by a single-exponential decay function. The points for steps #19 and 20 are not included because their decays were unable to be fitted by a single-exponential decay function.

S2.16 Energy calculations of the frameworks

We calculated the energies of **TK-COF-4/-5** and the hypothetical COFs with **bor** and **ctn** topology constructed by connecting **TFPT/TFPB** and **TAM**. The geometrical optimizations were conducted using COMPASS III force field with Materials Studio® software. Because the chemical formula and molar mass of these COFs are different, we calculate and show the energies in unit of kJ kg^{-1} so that the energies for different COFs are intercomparable. The chemical formula and molar mass of those COFs are as follows.

- **TK-COF-4**: chemical formula = $C_{196}H_{132}N_{28}$, molar mass = 2879.41 g mol^{-1}.
- **TK-COF-5/-5_dried**: chemical formula = $C_{208}H_{144}N_{16}$, molar mass = 2867.55 g mol^{-1}.
- Hypothetical COF with **bor** topology constructed from **TFPT** and **TAM**: chemical formula = $C_{171}H_{108}N_{24}$, molar mass = 2498.91 g mol^{-1}.
- Hypothetical COF with **ctn** topology constructed from **TFPT** and **TAM**: chemical formula = $C_{684}H_{432}N_{96}$, molar mass = 9995.65 g mol^{-1}.
- Hypothetical COF with **bor** topology constructed from **TFPB** and **TAM**: chemical formula = $C_{183}H_{120}N_{12}$, molar mass = 2487.06 g mol^{-1}.
- Hypothetical COF with **ctn** topology constructed from **TFPB** and **TAM**: chemical formula = $C_{732}H_{480}N_{48}$, molar mass = 9948.23 g mol^{-1}.

As summarized in Tables S9 and S10, the results revealed that the energies—especially non-bond energies—of **TK-COF-4** and **-5** are much lower than those of the hypothetical 3D-COF with **bor** and **ctn** topology, implying that **TK-COF-4** and **-5** are thermodynamically more stable than these hypothetical 3D-COFs.

Table S9. Energies calculated for **TK-COF-4** and the corresponding hypothetical **bor-** and **ctn-**topological COFs constructed by connecting **TFPT** and **TAM**

	This work	Hypothetical COFs	
	**TK-COF-4** [kJ kg^{-1}]	**bor** topology [kJ kg^{-1}]	**ctn** topology [kJ kg^{-1}]
**Valence energy (diag. terms): {A}**	651.53	772.41	785.53
Bond	68.14	85.79	71.69
Angle	883.58	122.70	227.49
Torsion	–307.30	563.04	485.35
Inversion	7.11	0.87	1.01
**Valence energy (cross terms): {B}**	–80.59	–4.68	–2.64
Stretch-Stretch	4.80	3.36	0.83
Stretch-Bend-Stretch	–9.94	–14.46	–22.90
Stretch-Torsion-Stretch	–24.20	–37.97	–21.78
Separated-Stretch-Stretch	3.69	4.46	1.67
Torsion-Stretch	–114.07	–158.42	–73.63
Bend-Bend	–0.67	0.00	0.00
Torsion-Bend-Bend	–0.90	0.43	–5.75
Bend-Torsion-Bend	60.69	157.92	118.92
**Non-bond energy: {C}**	–532.72	–16.27	65.63
van der Waals	75.10	254.78	329.64
Long Range Correction	–3.40	–0.58	–0.68
Electrostatic	–604.42	–270.47	–263.33
**Total energy = {A} + {B} + {C}**	38.22	711.47	848.52

Table S10. Energies calculated for **TK-COF-5** and the corresponding hypothetical **bor-** and **ctn-**topological COFs constructed by connecting **TFPB** and **TAM**

	This work		Hypothetical COFs	
	**TK-COF-5** [kJ kg^{-1}]	**TK-COF-5_dried** [kJ kg^{-1}]	**bor** topology [kJ kg^{-1}]	**ctn** topology [kJ kg^{-1}]
**Valence energy (diag. terms): {A}**	1148.14	1881.35	2160.75	2157.33
Bond	71.92	98.44	94.93	83.57
Angle	103.92	95.97	89.01	181.39
Torsion	962.47	1677.33	1974.79	1891.15
Inversion	9.83	9.61	2.02	1.22
**Valence energy (cross terms): {B}**	–84.41	–118.64	–122.82	–77.08
Stretch-Stretch	3.29	4.01	3.66	1.23
Stretch-Bend-Stretch	–5.90	–5.11	–5.39	–16.80
Stretch-Torsion-Stretch	–26.17	–48.95	–53.97	–35.66
Separated-Stretch-Stretch	0.56	–0.15	–1.47	–3.32
Torsion-Stretch	–111.71	–143.17	–138.87	–51.53
Bend-Bend	–0.14	–0.48	0.00	0.00
Torsion-Bend-Bend	–0.74	0.06	–0.50	–7.14
Bend-Torsion-Bend	56.41	75.15	73.73	36.13
**Non-bond energy: {C}**	–166.99	–226.86	240.77	317.12
van der Waals	54.08	–28.45	257.25	336.93
Long Range Correction	–3.71	–5.38	–0.58	–0.67
Electrostatic	–217.36	–193.02	–15.90	–19.13
**Total energy = {A} + {B} + {C}**	896.74	1535.85	2278.70	2397.36

Section S3. CO_2 adsorption properties reported for previous COFs, MOFs, and POPs

S3.1 COFs

The data used to construct Fig. 3g in the main text are summarized in Table S11 below.

Supplementary Table S11. Comparison of CO_2 adsorption properties reported for COFs

COF name	Q_{st} [kJ mol^{-1}]	IAST selectivity for CO_2/N_2 (15:85)		CO_2 uptake [cm^3 g^{-1}] at 100 kPa or 1 bar		Ref.
		273 K	298 K	273 K	298 K	
**TK-COF-4**	25.5	96.8	96.7	55.3	29.7	This work
**TK-COF-5**	25.3	78.4	85.4	45.4	26.3	
**Zn@CTF**	32.5	72	—	43.9	24.5	S13
**CTF-CSU38**	39.2	48.8	—	49.9	*33.6*	S14
**CTF-CSU39**	41.3	41.3	—	44.3	*41.2*	
**CTF-CSU40**	38.5	56.2	—	35.1	*24.9*	
**CTF-CSU41**	44.6	35.3	—	52.4	*40.2*	
**CTF-DI-2**	32.4	35	—	33.5	19.3	S15
**CTF-DI-3**	38.7	26	—	80.4	44.7	
**CTF-DI-4**	40.1	42	—	67.6	39.3	
**CTF-DI-5**	37.2	27	—	50.9	29.4	
**CTF-DI-6**	52.6	53	—	62.8	40	
**CTF-DI-7**	40.7	41	—	89.2	54.2	
**CTF-DI-8**	37.4	35	—	77.8	44.5	
**CTF-DI-9**	33.4	14	—	59.7	35.4	

**TpPa-NO2**	36.26	—	34.87	58.9	45.18	S16
**TMFPT-COF**	34.1	17	—	38.2	19.2	S17
**df-TzCTF400**	36.0	—	40	123.42	74.37	S18
**df-TzCTF600**	34.0	—	30	152.10	103.04	
**TzCTF400**	32.0	—	26	94.30	57.12	
**TzCTF600**	28.0	—	21	98.56	56.22	
**3D-TPB-COF-HQ**	23.5	—	40	93.4	62.8	S19
**3D-TPB-COF-Q**	29.0	—	93	105	75.9	
**3D-BMTA-COF-[Ac]$_{100\%}$**	29.0	78.9	—	54.4	36.4	S20
**3D-BMTA-COF-[Et]$_{100\%}$**	25.6	*33*	—	61.8	36.5	
**3D-BMTA-COF-[C≡CH]$_{100\%}$**	19.5	*13*	—	71.3	36.7	
**TBICOF**	42.8	46.7	40.3	68.89	39.04	S21
**NUS-2**	38.0	—	41.2	*84.0*	*51.52*	S22
**ACOF-1**	20.5	—	26.3	*53.8*	*32.5*	
**COF-300**	18.2	—	12.6	*30.9*	*22.8*	
**TAPB-PDA**	24.5	—	9.4	*30.2*	*15.7*	
**COF-LZU1**	22.4	—	31	*55.6*	*27.8*	
**TpPa-1**	44.0	—	113.9	*65.0*	*44.8*	
**Ru-COF**	36.0	64.2	—	88.35	61.5	S23

Numbers in italics are those read from the figures in the reference by the present authors.

S3.2 MOFs

To compare the CO_2/N_2 separation performance of **TK-COF-4**, which exhibited better performance than **TK-COF-5** in this report, with those reported for MOFs, we used the dataset of IAST CO_2/N_2 selectivity at 15:85 ratio ($S_{CN(15:85)}$) and Q_{st} in Table 2.3 of Ref. S24. For the data of **TK-COF-4**, we used $S_{CN(15:85)}$ (Fig. 3e in the main text) and $Q_{st(av)}$ (Fig. 3f in the main text). The result is shown in Fig. S35 below. Because the pressures at which $S_{CN(15:85)}$ were evaluated for the MOF reports were not indicated in the literature[S24], we have plotted both values of $S_{CN(15:85)}$ at 0 and 1 bar for **TK-COF-4** in this figure. According to this comparison, the present **TK-COF-4** has better performance than those MOFs in the sense that the former realized significantly low Q_{st} while retaining sufficiently high $S_{CN(15:85)}$ to be usable for CO_2 separation from most industrial flue gases. Note that the same conclusion is

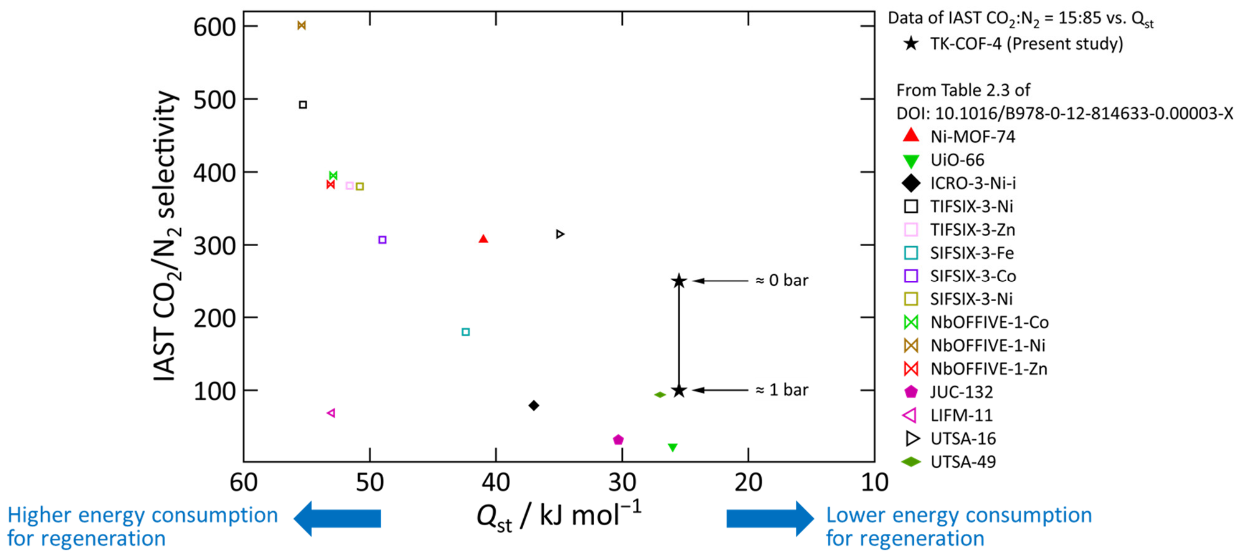

Supplementary Figure S35. Comparison of the values of IAST CO_2/N_2 selectivity at a 15:85 ratio and Q_{st} reported for previous MOFs presented in Table 2.3 of Ref. S24 and those for **TK-COF-4** presented in Figs. 3e and 3f in the main text, where $Q_{st} = Q_{st(av)}$.

drawn also when the dataset for previous MOFs compiled in Figure 2D of Ref. S25 is considered.

Next, we roughly compare the separation cost between **TK-COF-4** and a typical CO_2-separating MOF of **CALF-20**[S26]. **CALF-20** has similar CO_2/N_2 selectivity (≈ 100 @ 0.5 bar)[S26] to that of **TK-COF-4** (Fig. 3e in the main text). However, **CALF-20** has approximately twice the CO_2 adsorption capacity (3 mmol/g @ 25 °C)[S26] of **TK-COF-4** (30 cm^3 g^{-1} STP ≅ 1.35 mmol CO_2 g^{-1} @ 298 K, Fig. 3d in the main text). Although, rigorously speaking, working capacity and adsorption capacity are different, we conduct here a coarse estimation for the purpose of comparison. According to Figure 2.2 of Ref. S24 and Figure 7 of Ref. S27, for the CO_2/N_2 selectivity of 100, the twofold difference in the CO_2 adsorption capacity causes only a minor difference (*ca.* 7 %) in the separation cost. However, the heat of adsorption of CO_2 of **CALF-20** (*ca.* 38 kJ mol^{-1})[S26] is 50% higher than that of **TK-COF-4** (*ca.* 25 kJ mol^{-1}), the difference of which shall directly impact the energy cost required for the regeneration process. Therefore, the CO_2/N_2 separation cost of **TK-COF-4** is considered to be lower in terms of energy required for the regeneration and slightly higher in terms of CO_2 adsorption capacity than **CALF-20**.

S3.3 POPs

The CO_2/N_2 separation performance of **TK-COF-4**, which exhibited better performance than **TK-COF-5** in this report, is compared with those reported for porous organic polymers (POPs). For the data of **TK-COF-4**, we used the IAST CO_2/N_2 selectivity at a 15:85 ratio ($S_{CN(15:85)}$) and $Q_{st(av)}$, shown in Figs. 3e and 3f in the main text, respectively. For the data of POPs, we used the data shown in Table 1 of Ref. S28, for which the values of $S_{CN(15:85)}$ and Q_{st} were reported. The result is shown in Fig. S36 below. Because the pressures at which $S_{CN(15:85)}$ were evaluated for the POP reports were often not clearly indicated in the literature, we have plotted the values of $S_{CN(15:85)}$ at both 0 and 1 bar for **TK-COF-4** in this figure. According to this comparison, the present **TK-COF-4** has better performance generally than those POPs in the sense that the former realized rather low Q_{st} while retaining sufficiently high $S_{CN(15:85)}$ for CO_2 separation from most industrial flue gases.

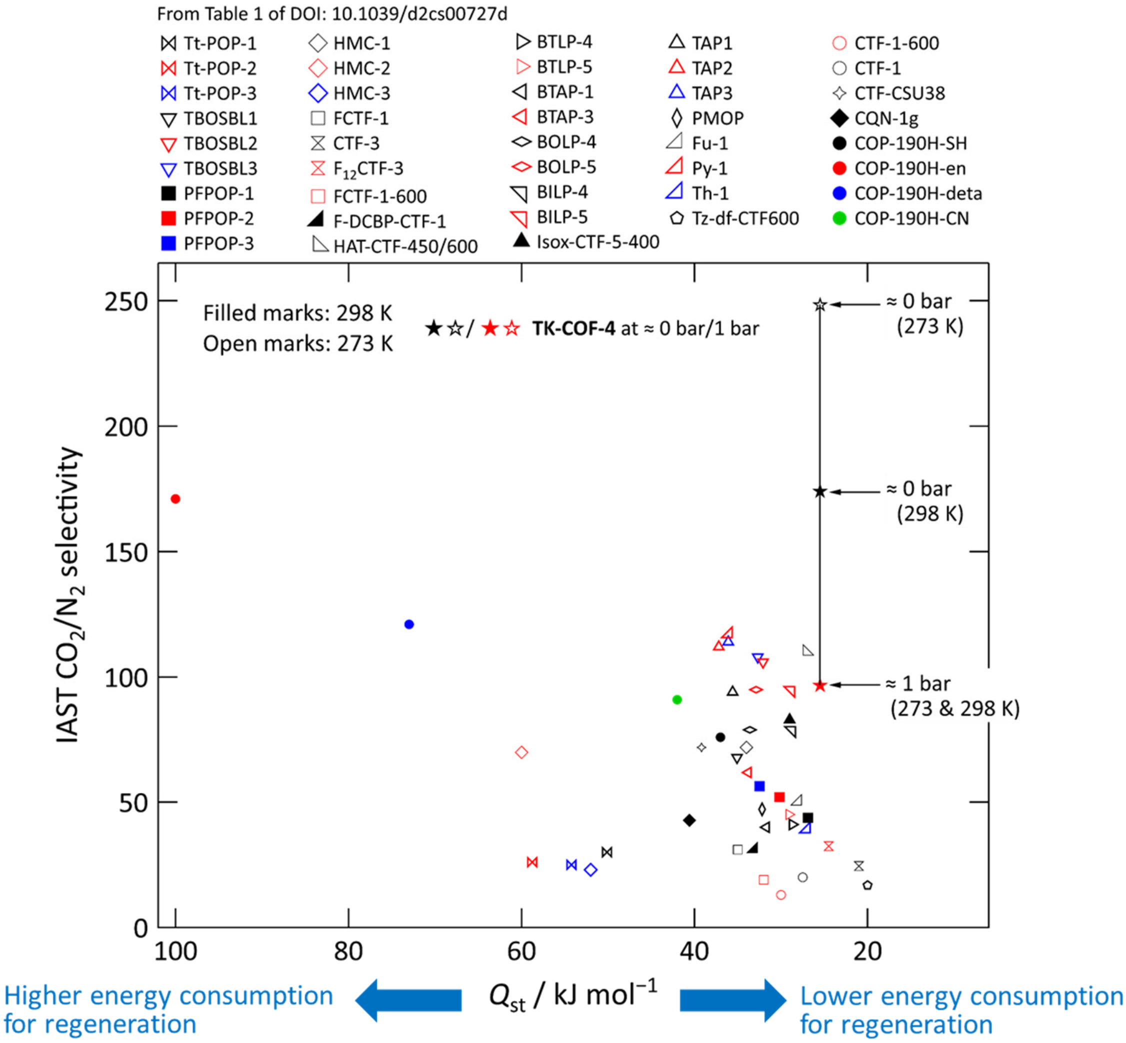

Supplementary Figure S36. Comparison of the values of IAST CO_2/N_2 selectivity at a 15:85 ratio and Q_{st} reported for previous POPs presented in Table 1 of Ref. S28 and those for **TK-COF-4** presented in Figs. 3e and 3f in the main text, where $Q_{st} = Q_{st(av)}$.

Section S4. Explanation for A- and B-level alerts in checkCIF report

Validation reply for TK-COF-4 (CCDC number 2361003):

```
# start Validation Reply Form
_vrf_PLAT084_TK-COF-4
;
PROBLEM: High wR2 Value (i.e. > 0.25) ..................      0.37 Report
RESPONSE: Due to solvent disorder.
;
_vrf_PLAT230_TK-COF-4
;
PROBLEM: Hirshfeld Test Diff for    C19      --C20      .       8.6 s.u.
RESPONSE: Due to large thermal motions of C19 and C20.
;
_vrf_PLAT934_TK-COF-4
;
PROBLEM: Number of (Iobs-Icalc)/Sigma(W) > 10 Outliers ..         7 Check
RESPONSE: Due to solvent disorder.
;
```

Validation reply for TK-COF-5 (CCDC number 2361014):

```
# start Validation Reply Form
_vrf_PLAT084_TK-COF-5
;
PROBLEM: High wR2 Value (i.e. > 0.25) ..................      0.41 Report
RESPONSE:Due to solvent disorder.
;
_vrf_PLAT934_TK-COF-5
;
PROBLEM: Number of (Iobs-Icalc)/Sigma(W) > 10 Outliers ..         9 Check
RESPONSE:Due to solvent disorder.
;
```

Validation reply for TK-COF-5_dried (CCDC number 2383526):

start Validation Reply Form
_vrf_RINTA01_TK-COF-5_dried
;
PROBLEM: The value of Rint is greater than 0.18
RESPONSE: The diffraction intensity was generally weak because the crystallinity decreased after solvent desorption.
;
_vrf_PLAT026_TK-COF-5_dried
;
PROBLEM: Ratio Observed / Unique Reflections (too) Low .. 22% Check
RESPONSE: The diffraction intensity was generally weak because the crystallinity decreased after solvent desorption.
;
_vrf_PLAT084_TK-COF-5_dried
;
PROBLEM: High wR2 Value (i.e. > 0.25) .... 0.56 Report
RESPONSE: The diffraction intensity was generally weak because the crystallinity decreased after solvent desorption.
;
_vrf_PLAT234_TK-COF-5_dried
;
PROBLEM: Large Hirshfeld Difference C00Z --C017 . 0.32 Ang.
RESPONSE: In the crystals after solvent desorption, the molecules were loosely packed and both atoms were analyzed with large ADP, but these atomic species are correct.
;
_vrf_PLAT602_TK-COF-5_dried
;
PROBLEM: Solvent Accessible VOID(S) in Structure .... ! Check
RESPONSE: The crystals after solvent desorption showed a void with a volume of 1081.70A^3 (23.5%) in the unit cell, but no significant residual electron density was observed in the void.
;
_vrf_PLAT020_TK-COF-5_dried
;
PROBLEM: The Value of Rint is Greater Than 0.12 .... 0.213 Report
RESPONSE: The diffraction intensity was generally weak because crystallinity decreased after solvent desorption.
;
_vrf_PLAT082_TK-COF-5_dried
;
PROBLEM: High R1 Value .... 0.18 Report

RESPONSE: The diffraction intensity was generally weak because the crystallinity decreased after solvent desorption.
;
_vrf_PLAT241_TK-COF-5_dried
;
PROBLEM: High 'MainMol' Ueq as Compared to Neighbors of C01B Check
RESPONSE: ADP of some carbon atoms (C01B, C01C, C01E, C01J, and C01K) was analyzed with large ADP due to the libration of the benzene ring, which was amplified by the lowered crystallinity after solvent desorption. Due to the lowering of crystallinity after solvent desorption, the carbon atom of C01G was analyzed to have large ADP values.
;
_vrf_PLAT242_TK-COF-5_dried
;
PROBLEM: Low 'MainMol' Ueq as Compared to Neighbors of C002 Check
RESPONSE: ADP of some carbon atoms was analyzed with large ADP due to the libration of the benzene ring, which was enhanced by the lowered crystallinity after solvent desorption.
;
_vrf_PLAT334_TK-COF-5_dried
;
PROBLEM: Small <C-C> Benzene Dist. C002 -C01H . 1.32 Ang.
RESPONSE: ADP of some carbon atoms was large due to the libration of the benzene ring. This resulted in shorter C-C distances.
;
_vrf_PLAT340_TK-COF-5_dried
;
PROBLEM: Low Bond Precision on C-C Bonds .... 0.02274 Ang.
RESPONSE: In the crystals after solvent desorption, the molecules were loosely packed and atoms were analyzed with large ADP. This resulted in lower accuracy of C-C bond lengths.
;
_vrf_PLAT410_TK-COF-5_dried
;
PROBLEM: Short Intra H...H Contact H012 ..H01K . 1.81 Ang.
RESPONSE: Due to the large libration of the benzene ring, the carbon and hydrogen atoms are analyzed as average positions and appear to be close to each other.
;
end Validation Reply Form

References

S1. Uribe-Romo, F. J., Hunt, J. R., Furukawa, H., Klöck, C., O'Keeffe, M. & Yaghi, O. M. A crystalline imine-linked 3-D porous covalent organic framework. *J. Am. Chem. Soc.* **131**, 4570–4571 (2009).

S2. Stegbauer, L., Schwinghammer, K. & Lotsch, B. V. A hydrazone-based covalent organic framework for photocatalytic hydrogen production. *Chem. Sci.* **5**, 2789–2793 (2014).

S3. Ma, T., Kapustin, E. A., Yin, S. X., Liang1, L., Zhou, Z., Niu, J., Li, L.-H., Wang, Y., Su, J., Li, J., Wang, X., Wang, W. D., Wang, W., Sun, J. & Yaghi, O. M. Single-crystal x-ray diffraction structures of covalent organic frameworks. *Science* **361**, 48–52 (2018).

S4. Patra, B. C., Das, S. K., Ghosh, A., K, A. R. Moitra, P., Addicoat, M., Mitra, S., Bhaumik, A., Bhattacharya, S. & Pradhan, A. Covalent organic framework based microspheres as an anode material for rechargeable sodium batteries. *J. Mater. Chem. A* **6**, 16655–16663 (2018).

S5. Grunenberg, L., Savasci, G., Terban, M. W., Duppel, V., Moudrakovski, I., Etter, M., Dinnebier, R. E., Ochsenfeld, C. & Lotsch, B. V. Amine-linked covalent organic frameworks as a platform for postsynthetic structure interconversion and pore-wall modification. *J. Am. Chem. Soc.* **143**, 3430−3438 (2021).

S6. Banerjee, T., Haase, F., Trenker, S., Biswal, B. P., Savasci, G., Duppel, V., Moudrakovski, I., Ochsenfeld, C. & Lotsch, B. V. Sub-stoichiometric 2D covalent organic frameworks from tri- and tetratopic linkers. *Nature Commun.* **10**, 2689 (2019).

S7. Tang, X., Yang, Y., Li, X., Wang, X., Guo, D., Zhang, S., Zhang, K., Wu, J., Zheng, J., Zheng, S., Fan, J., Zhang, W. & Cai, S. Postmodification of an amine-functionalized covalent organic framework for enantioselective adsorption of tyrosine. *ACS Appl. Mater. Interfaces* **15**, 24836–24845 (2023).

S8. Lyu, H., Li, H., Hanikel, H., Wang, K. & Yaghi, O. M. Covalent organic frameworks for carbon dioxide capture from air. *J. Am. Chem. Soc.* **144**, 12989−12995 (2022).

S9. Dautzenberg, E., Li, G. & de Smet, L. C. P. M. Aromatic amine-functionalized covalent organic frameworks (COFs) for CO_2/N_2 separation. *ACS Appl. Mater. Interfaces* **15**, 5118−5127 (2023).

S10. Han, X., Zhou, Z., Wang, K., Zheng, Z., Neumann, S. E., Zhang, H., Ma, T. & Yaghi, O. M. Crystalline polyphenylene covalent organic frameworks. *J. Am. Chem. Soc.* **146**, 89−94 (2024).

S11. Zhou, Z., Ma, T., Zhang, H., Chheda, S., Li, H., Wang, K., Ehrling, S., Giovine, R., Li, C., Alawadhi, A. H., Abduljawad, M. M., Alawad, M. O., Gagliardi, L., Sauer, J. & Yaghi, O. M. Carbon dioxide capture from open air using covalent organic frameworks. *Nature* (2024). (DOI: https://doi.org/10.1038/s41586-024-08080-x)

S12. Srikanth, C. S. & Chuang, S. S. C. Spectroscopic investigation into oxidative degradation of silica-supported amine sorbents for CO_2 capture. *ChemSusChem* **5**, 1435–1442 (2012).

S13. Guo, B., Wu, C., Su, Q., Liu, Z., Li, X., Li, G. & Wu, Q. A Zn-salen based covalent triazine framework as a promising candidate for CO_2 capture. *Mater. Lett.* **221**, 236–239 (2018).

S14. Fu, Y., Wang, Z., Li, S., He, X., Pan, C., Yan, J. & Yu, G. Functionalized covalent triazine frameworks for effective CO_2 and SO_2 removal. *ACS Appl. Mater. Interfaces* **10**, 36002−36009 (2018).

S15. Dua, J., Cuia, Y., Liua, Y., Krishnac, R., Yu, Y., Wanga, S., Zhanga, C., Songa, X. & Liang, Z. Preparation of benzodiimidazole-containing covalent triazine frameworks for enhanced selective CO_2 capture and separation. *Microporous Mesoporous Mater.* **276**, 213–222 (2019).

S16. Xiong, X.-H., Zhang, L., Wang, W., Zhu, N.-X., Qin, L.-Z., Huang, H.-F., Meng, L.-L., Xiong, Y.-Y., Barboiu, M., Fenske, D., Hu, P. & Wei, Z.-W. Nitro-decorated microporous covalent organic framework (TpPa-NO_2) for selective separation of C_2H_4 from a C_2H_2/C_2H_4/CO_2 mixture and CO_2 capture. *ACS Appl. Mater. Interfaces* **14**, 32105−32111 (2022).

S17. Li, X., Su, Q., Luo, K., Li, H., Li, G. & Wu, Q. Construction of a highly heteroatom-functionalized covalent organic framework and its CO_2 capture capacity and CO_2/N_2 selectivity. *Mater. Lett.* **282**, 128704 (2021).

S18. Mukherjee, S., Das, M., Manna, A., Krishna, R. & Das, S. Newly designed 1,2,3-triazole functionalized covalent triazine frameworks with exceptionally high uptake capacity for both CO_2 and H_2. *J. Mater. Chem. A* **7**, 1055–1068 (2019).
S19. Gao, C., Li, J., Yin, S., Sun, J. & Wang, C. Redox-triggered switching in three-dimensional covalent organic frameworks. *Nat. Commun.* **11**, 4919 (2020).
S20. Gui, B., Liu, X., Cheng, Y., Zhang, Y., Chen, P., He, M., Sun, J. & Wang, C. Tailoring the pore surface of 3D covalent organic frameworks via post-synthetic click chemistry. *Angew. Chem. Int. Ed.* **61**, e202113852 (2022).
S21. Das, P. & Mandal, S. K. In-depth experimental and computational investigations for remarkable gas/vapor sorption, selectivity, and affinity by a porous nitrogen-rich covalent organic framework. *Chem. Mater.* **31**, 1584−1596 (2019).
S22. Wang, Y., Kang, C., Zhang, Z., Usadi, A. K., Calabro, D. C., Baugh, L. S., Yuan, Y. D. & Zhao, D. Evaluation of Schiff-base covalent organic frameworks for CO_2 capture: structure−performance relationships, stability, and performance under wet conditions. *ACS Sustainable Chem. Eng.* **10**, 332−341 (2022).
S23. Kumar, G., Singh, M., Goswami, R. & Neogi, S. Structural dynamism–actuated reversible CO_2 adsorption switch and postmetalation-induced visible light Cα–H photocyanation with rare size selectivity in N-functionalized 3D covalent organic framework. *ACS Appl. Mater. Interfaces* **12**, 48642−48653 (2020).
S24. Mukherjee, S., Kumar, A. & Zaworotko, M. J. Metal-organic framework based carbon capture and purification technologies for clean environment. Chapter 2 of *Metal-Organic Frameworks (MOFs) for Environmental Applications (Edited by Ghosh, S. K.)*, Elsevier, Amsterdam, 2019. DOI: 10.1016/B978-0-12-814633-0.00003-X
S25. Li, Y., Bai, Y., Wang, Z., Gong, Q., Li, M., Bo, Y., Xu, H., Jiang, G. & Chi, K. Exquisitely constructing a robust MOF with dual pore sizes for efficient CO_2 capture. *Molecules* **28**, 6276 (2023).
S26. Nguyen, T. T. T., Lin, J. -B., Shimizu, G. K. H. & Rajendran, A. Separation of CO_2 and N_2 on a hydrophobic metal organic framework CALF-20. *Chem. Eng. J.* **442**, 136263 (2022).

S27. Ho, M. T., Allinson, G. W. & Wiley, D. E. Reducing the cost of CO_2 capture from flue gases using pressure swing adsorption. *Ind. Eng. Chem. Res.* **47**, 4883−4890 (2008).
S28. Song, K. S., Fritz, P. W. & Coskun, A. Porous organic polymers for CO_2 capture, separation and conversion. *Chem. Soc. Rev.* **51**, 9831−9852 (2022).
